# Specific Inflammatory Stimuli Lead to Distinct Platelet Responses in Mice and Humans

**DOI:** 10.1371/journal.pone.0131688

**Published:** 2015-07-06

**Authors:** Lea M. Beaulieu, Lauren Clancy, Kahraman Tanriverdi, Emelia J. Benjamin, Carolyn D. Kramer, Ellen O. Weinberg, Xianbao He, Samrawit Mekasha, Eric Mick, Robin R. Ingalls, Caroline A. Genco, Jane E. Freedman

**Affiliations:** 1 University of Massachusetts Medical School, Department of Medicine, Division of Cardiovascular Medicine, Worcester, MA, United States of America; 2 NHLBI and Boston Universitys Framingham Heart Institute, Framingham, MA, United States of America; 3 Boston University School of Medicine, Department of Medicine, Cardiology and Preventive Medicine Section, Boston, MA, United States of America; 4 Boston University School of Medicine, Department of Medicine, Boston, MA, United States of America; 5 Boston University School of Medicine, Department of Medicine, Section of Infectious Diseases, Boston, MA, United States of America; 6 Boston Medical Center, Boston, MA, United States of America; 7 University of Massachusetts Medical School, Department of Quantitative Health Sciences, Worcester, MA, United States of America; 8 Boston University School of Medicine, Department of Microbiology, Boston, MA, United States of America; Medical Faculty, Ludwig Maximilians University Munich, GERMANY

## Abstract

**Introduction:**

Diverse and multi-factorial processes contribute to the progression of cardiovascular disease. These processes affect cells involved in the development of this disease in varying ways, ultimately leading to atherothrombosis. The goal of our study was to compare the differential effects of specific stimuli – two bacterial infections and a Western diet – on platelet responses in ApoE^-/-^ mice, specifically examining inflammatory function and gene expression. Results from murine studies were verified using platelets from participants of the Framingham Heart Study (FHS; n = 1819 participants).

**Methods:**

Blood and spleen samples were collected at weeks 1 and 9 from ApoE^-/-^ mice infected with *Porphyromonas gingivalis* or *Chlamydia pneumoniae* and from mice fed a Western diet for 9 weeks. Transcripts based on data from a Western diet in ApoE^-/-^ mice were measured in platelet samples from FHS using high throughput qRT-PCR.

**Results:**

At week 1, both bacterial infections increased circulating platelet-neutrophil aggregates. At week 9, these cells individually localized to the spleen, while Western diet resulted in increased platelet-neutrophil aggregates in the spleen only. Microarray analysis of platelet RNA from infected or Western diet-fed mice at week 1 and 9 showed differential profiles. Genes, such as Serpina1a, Ttr, Fgg, Rpl21, and Alb, were uniquely affected by infection and diet. Results were reinforced in platelets obtained from participants of the FHS.

**Conclusion:**

Using both human studies and animal models, results demonstrate that variable sources of inflammatory stimuli have the ability to influence the platelet phenotype in distinct ways, indicative of the diverse function of platelets in thrombosis, hemostasis, and immunity.

## Introduction

Cardiovascular disease (CVD) is a chronic inflammatory process with multiple etiologies, including genetic and environmental factors. Bacterial infections and obesity are two known pro-inflammatory contributors to the development of CVD. The effects of these inflammatory stimuli on vascular cells have been extensively studied, with the exception for the second most abundant cell in circulation, the platelet. Particularly, in light of work which shows the relationship between platelet transcripts and CVD in FHS [1,2] and their new role in inflammation [3,4,5], questions still remain as to how platelets contribute to the development of CVD. The goals of our study were to characterize the differential effects of two common bacterial infections and a Western diet on platelet inflammatory function and gene transcripts in ApoE^-/-^ mice and in human samples. These results will help to better understand the connections between inflammatory stimuli, cardiovascular disease, and platelets.

One bacterium associated with CVD is the gram-negative oral pathogen *Porphyromonas gingivalis* (*P*. *gingivalis*), which induces low-grade chronic inflammation associated with oral bone loss, clinically known as periodontal disease. This bacterium expresses several outer membrane proteins which are recognized by the innate immune system through Toll-like receptor 2 (TLR2). In ApoE^-/-^ mice, *P*. *gingivalis* accelerates atherosclerotic plaque size [6,7,8], with accumulation of lipids, T cells, and macrophages compared to uninfected mice [7]. Plaque collagen and elastin content is also altered with infection, suggesting that *P*. *gingivalis* infection affects the stability of atherosclerotic plaques [7]. In humans, *P*. *gingivalis* is detected in atherosclerotic plaques [9,10] and is associated with the development of carotid artery atherosclerosis [11].

Another bacterium associated with CVD is the obligate intracellular gram-negative bacterium, *Chlamydia pneumoniae* (*C*. *pneumoniae*). It is associated with atypical pneumonia, as well as pharyngitis, bronchitis, and sinusitis in humans. This bacterium is also recognized in part by TLR2 on innate immune cells. It has also been demonstrated to increase atherosclerotic plaque size, lipid content, and dendritic cells in ApoE^-/-^ mice compared to uninfected mice [12]. In humans, *C*. *pneumoniae* antibody seropositivity is associated with the progression of coronary artery calcification [13] and risk of coronary heart disease and myocardial infarction[14]. *C*. *pneumoniae* has also been identified through PCR methodology in atherosclerotic plaques in humans [15,16].

Obesity, having a body mass index (BMI) ≥30 kg/m^2^, is another key risk factor associated with CVD. In the US, 35% of adults are considered obese [17]. For individuals ≥20 years of age surveyed between 2009 and 2010, 35.8% were obese and 72.5% had a health score of 0–1, classifying them in poor cardiovascular health [17]. Consistent with the importance of weight and diet, in animal models of atherosclerosis, feeding animals a high fat diet increases atherosclerotic plaque size, number, and severity [18,19,20].

We have previously demonstrated that in human and mouse platelets, *P*. *gingivalis* is recognized by TLR2 and induces aggregation [21] and heterotypic aggregate formation [3,4]. *C*. *pneumoniae* also binds to platelets to induce aggregation and P-selectin expression [22]. A Western diet in C57BL/6J mice increases thrombin-induced platelet adhesion [3]. The goal of this study was to characterize the differential effects of these two common bacterial infections and a Western diet on platelet inflammatory function and gene transcripts at different timepoints in a well-established atherosclerosis murine model prior to the formation of plaques. These results were also correlated with recent observations from aortic tissue following exposure to the same inflammatory stimuli. [23]. Our results suggest that both inflammatory stimuli and the kinetics of the response differentially influence platelet transcripts and inflammatory function. Furthermore, our results were corroborated in human studies and provide insight into how platelets alter the development of CVD.

## Materials and Methods

### Animals

Mice were cared for at the Boston University School of Medicine (BUSM) Vivarium. All work was approved by BUSM Institutional Animal Care and Use Committee. Mice were housed in a 12 hour light cycle at constant temperature and humidity with free access to food and water. During procedures, mice were monitored and removed if exhibiting signs of distress. *P*. *gingivalis* strain 381 and *C*. *pneumoniae* strain AO3 were grown and purified as previously described [24,25]. One set of ApoE^-/-^ male mice (Jackson Laboratories) were treated with 4% sulfamethoxazole in their drinking water for 10 days to clear normal oral microflora [23]. These mice were then challenged 5 times per week for 3 weeks on the buccal surface of their maxilla with 1x10^9^ CFU *P*. *gingivalis* in 2% carboxymethyl cellulose. An additional set of ApoE^-/-^ male mice were challenged intranasally once per week for three weeks with 2x10^6^ IFU *C*. *pneumoniae* suspended in sucrose-phosphate-glutamate buffer while under light anesthesia of ketamine and xylazine [23]. Mice were sacrificed by CO_2_ asphyxiation followed by cervical dislocation at an early timepoint 1 day after the last *P*. *gingivalis* challenge (~9 weeks of age at start) and 4 days after the last *C*. *pneumoniae* exposure (~9 weeks of age at start), with n = 3 for each group [23]. Another group was sacrificed 9 weeks later (~6 weeks of age at start for both groups), with n = 3 for each group, prior to the formation of overt aortic plaque formation as seen at later timepoints with these stimuli [7,12,23,26]. A final group of ApoE^-/-^ male mice (~16 weeks of age at start) were fed a Western diet (17.3% protein, 48.5% carbohydrates, 1.2% fat, and 0.2% cholesterol; Teklad Harlan) for 9 weeks and then sacrificed with n = 6 for the Western diet [23]. Analysis was performed on samples collected from mice at each timepoint.

### Whole Blood Cell and Platelet Counts

Blood collected from each mouse was analyzed for white blood cell (WBC) and platelet content using a COULTER A^C.^T Series Analyzer (Becton Dickinson) [3].

### Heterotypic Aggregate Formation

A sample of whole blood from each mouse was dual stained with anti-mouse CD41 FITC-conjugated and anti-mouse Ly6G PE-Cy7-conjugated antibodies and appropriate isotype controls (eBioscience; Cat# 11–0411 and 25–5931, respectively). The percent of platelet-positive neutrophils was determined using a FACSCalibur Flow Cytometer with Cell Quest Software (BD Bioscience) [3,4].

### Spleen Sectioning and Staining

Frozen spleen sections were stained with anti-mouse CD41 (Abcam; Cat# ab63983) and anti-mouse Ly6G and Ly6C (BD Bioscience; Cat# 553123) antibodies and appropriate isotype controls, followed by anti-rabbit IgG Alexa Fluor 405-conjugated and anti-rat Texas Red-X secondary antibodies (Life Technologies; Cat# A-31556 and T-6392, respectively). Samples were visualized using Leica TCS SP5 II Confocal Microscope with Leica Application Suite Advanced Fluorescence Version 2.6.0.7266 software then analyzed with ImageJ (v1.46r) and Adobe Photoshop CS3 (v13.0.1).

### Platelet RNA Isolation

Platelets were isolated from blood samples using a series of centrifugation steps. Briefly, samples were centrifuged at 450*xg* for 7 minutes. The top layer consisting of platelet rich plasma (PRP) was removed and diluted 2.33X with platelet wash buffer (10 mM sodium citrate, 150 mM sodium chloride, 1 mM EDTA, 1% dextrose, pH 7.4) and 1:10000 prostaglandin E_1_ (Calbiochem). Samples were centrifuged at 300*xg* for 4 minutes to remove any contaminating WBC. The supernatant was further diluted 3X with platelet wash buffer and PGE_1,_ then centrifuged at 3500*xg* for 10 minutes [3]. The resulting platelet pellet was lysed and RNA was isolated using the miRNeasy Kit (Qiagen).

### RNA Microarray and Analysis

RNA from each mouse in each condition was pooled to generate at least 50 ng of RNA per sample. Samples were reverse transcribed using Ovation Pico WTA System V2 (Nugen) and purified using Agencourt RNA Clean XP Purification Beads. After SPIA amplification and purification, samples were labeled using the Encore Biotin Module (Nugen) and hybridized to the GenChip Mouse Gene 1.0 ST Array (Affymetrix), stained, and scanned using an Affymetrix GeneArray Scanner 3000 7G Plus. All procedures were performed by the Microarray Resource Facility at Boston University. Affymetrix CEL files were normalized to produce gene-level expression values using the Robust Multiarray Average (RMA) [27] in the Affymetrix Bioconductor (v1.28.1) [28] and an Entrez Gene-specific probe set (v14.0.0) mapping from the Molecular Behavioral Neuroscience Institute at the University of Michigan (http://brainarray.mbni.med.umich.edu/Brainarray/Database/CustomCDF) [29]. Array quality was assessed by computing Relative Log Expression (RLE) and Normalized Unscaled Standard Error (NUSE) using the AffyPLM Bioconductor (v1.26.1) [30]. All microarray analyses were performed using the R environment (v2.15.1) for statistical computing. Gene Set Enrichment Analysis (GSEA; v2.0.13) [31] was used to identify biological terms, pathways, and processes. By using the normalized enrichment score (NES) of ≥2/-2, the false discovery rate (FDR *q*) of <0.05, and the nominal *p-*value (Nom *p*) of <0.05, we identified gene sets that were significantly enriched for and would have been affected the most by treatment. The Entrez Gene identifiers of the mouse homologs of the genes (as determined using HomoloGene v65 [32]) were ranked according to the log2-transformed fold change computed for each comparison; in cases where a given mouse gene mapped to more than one human gene, one homolog was randomly chosen. These ranked lists were then used to perform pre-ranked GSEA analyses (default parameters with random seed 1234) using Entrez Gene versions of the Biocarta, KEGG, Reactome, and Gene Ontology (GO) gene sets obtained from the Molecular Signature Database (MsigDB; v3.0) [33]. Post analysis information on transcript alternative names and functions were determined using genecard.org and ncbi.nlm.nih.gov. Heatmaps were created using MeV v4.9.0 [34].

### qRT-PCR Verification

Isolated RNA was converted to cDNA using High-Capacity cDNA Reverse Transcription Kit (Life Technologies), followed by pre-amplification using TaqMan PreAmp Master Mix (Life Technologies). qRT-PCR was performed on the Applied Biosystems 7900 HT Fast Real-Time PCR System with SDS 2.2.2 Software (Life Technologies) using primers and probes for the following genes (Life Technologies): microfibrillar-associated protein 1a (MFAP1A), glyceraldehyde-3-phosphate dehydrogenase (GAPDH), β-actin (ACTB), and β2-microglobulin (B2M). Analysis included correcting by the mean of the three housekeeping genes (ΔCt)–GAPDH, ACTB, and B2M – followed by calculating the difference between treatment and Untreated Controls (ΔΔCt). Fold change (graphed) was then determined by calculating 2^-ΔΔCt^.

### Framingham Heart Study

As previously described [1,2,3], platelet RNA was isolated from 1819 participants of the FHS Offspring Cohort Examination 8 using miRNeasy kit (Qiagen) and converted to cDNA. Pre-amplification was performed using TaqMan PreAmp Master Mix (Life Technologies). Quantitative RT-PCR was performed using the BioMark System (Fluidigm) with primers and probes from Life Technologies. Results were compared to data collected from the medical history and physical examination of each participant. Individuals were categorized as having hypertension if they had systolic blood pressure ≥140 mm Hg, diastolic blood pressure ≥90 mm Hg, or were receiving treatment for hypertension. They were categorized as having diabetes if their fasting blood glucose was ≥126 mg/dL, their non-fasting blood glucose was ≥200 mg/dL, or were being treated with insulin or hypoglycemic drugs. Finally, individuals were categorized as smokers if they had smoked 1 cigarette a day in the year prior to examination. All of this work with the FHS was approved by the University of Massachusetts Medical School and Boston University Medical Center Institutional Review Boards and all participants gave written informed consent [1,2,3]. Interleukin 6 (IL6) and C-reactive protein (CRP) levels were measured as previously described [2].

### Statistics

Data from ApoE^-/-^ mouse studies were reported as the mean ± the standard deviation. In some cases, the percent compared to Untreated Controls, which was set to 100%, was reported. All data were analyzed by ANOVA followed by Tukey’s Multiple Comparison Test using GraphPad Prism 5 software. Data were considered normally distributed, with a two-sided *p*<0.05 being significant.

For the FHS, descriptive statistics were reported as the mean ± standard deviation for continuous variable or the count (percent) for binary variables. Quantitative RT-PCR cycle threshold (Ct) values were corrected (ΔCT) by the geometric mean of three housekeeping genes–GAPDH, ACTB, B2M. Genes not expressed and failing to report a Ct value within a set period were assigned the maximum Ct value allowed in our procedure (28 cycles). Multivariable linear regression models for gene expression (ΔCt) were fitted adjusting for the following confounders–BMI, smoking status, total cholesterol, HDL cholesterol, triglycerides, systolic blood pressure, diastolic blood pressure, glucose levels, diabetes, coronary heart disease [angina pectoris, coronary insufficiency, myocardial infarction adjudicated by a review panel], lipid-lowering therapy, hormone replacement therapy, antihypertensive therapy, and regular aspirin therapy (at least 3 times a week). All statistical analysis was performed using Stata 12.0 software [1,2,3]. Statistical significance was assessed using two-sided *p*-value, with those <0.05 being considered statistically significant.

## Results

### Differential Platelet Gene Expression in ApoE^-/-^ Mice Infected with Bacteria or Fed a Western Diet

**Fig 1:**
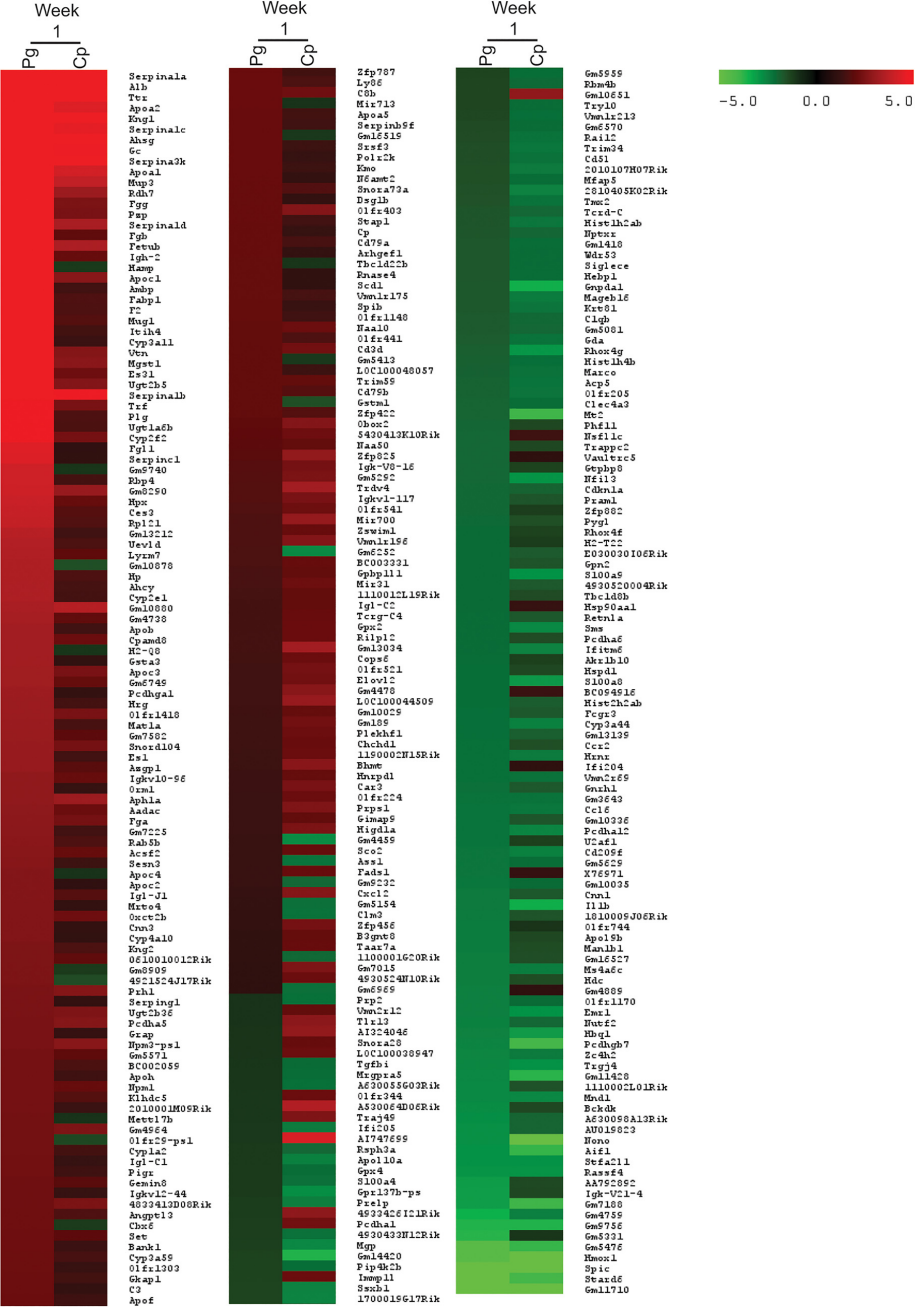
Genes upregulated and downregulated at week 1 in ApoE^-/-^ mice with bacterial infection. Heatmap shows the transcripts identified through microarray as upregulated or downregulated 2-fold or more with either *P*. *gingivalis* (Pg) or *C*. *pneumoniae* (Cp) infection compared to Untreated Control in the ApoE^-/-^ mice. Each condition represents RNA from 3 mice pooled.

ApoE^-/-^ mice (n = 3) were infected orally with *P*. *gingivalis* or intranasally with *C*. *pneumoniae*. One day after the last oral infection with *P*. *gingivalis* and 4 days after the last intranasal infection with *C*. *pneumoniae* (week 1), platelet RNA was isolated and pooled to generate a sufficient amount of RNA per condition for microarray analysis. Due to the small sample size, only genes up- or downregulated 2-fold or greater and pathways with a normalized enrichment score (NES) ≥ ~2/-2, nominal *p*-value (Nom *p*) <0.05, and a false discovery rate (FDR *q*) <0.05 were considered. Additionally, this data was intended to generate hypotheses on how platelets and platelet transcripts are affected by different inflammatory stimuli over time. During Week 1, we observed increased expression of 129 genes and decreased expression in 59 genes following *P*. *gingivalis* infection compared to Untreated Controls (Fig 1). The top five upregulated genes with known function were SERPINA1A (27.09-fold), ALB (17.83-fold), TTR (15.84-fold), APOA2 (15.08-fold), and KNG1 (13.01-fold). The top 5 downregulated genes were STARD6 (-5.49-fold), SPIC (-5.42-fold), HMOX1 (-4.60-fold), IGK-V21-4 (-3.08-fold), and RASSF4 (-2.89-fold). Using Gene Set Enrichment Analysis (GSEA), we identified gene sets with increased expression following *P*. *gingivalis* infection with an NES ≥2, a Nom *p* <0.05, and an FDR *q* <0.05. These gene sets included coagulation, lipids, signaling pathways, RNA/gene expression, and inflammation (S1 Table). Gene sets, which exhibited decreased expression, included those involved in enzyme activity, differentiation, metabolism, extracellular matrix related, signaling, and cytoskeleton pathways; however, these gene sets were not significant (NES ˃-2, FDR *q* ˃0.05; S2 Table).

In ApoE^*-/-*^ mice infected with *C*. *pneumoniae* at week 1, there were 87 platelet transcripts increased and 70 transcripts decreased 2-fold or more compared to Untreated Control (Fig 1). The top 5 genes that exhibited an increase in expression were SERPINA1A (13.17-fold), ALB (7.75-fold), SERPINA3K (6.47-fold), TTR (6.20-fold), and KNG1 (5.14-fold). The top 5 genes that exhibited a decrease in expression were NONO (-10.89-fold), SPIC (-6.92-fold), HMOX1 (-5.15-fold), PCDHGB7 (-4.09-fold), and STARD6 (-4.09-fold). GSEA identified the following positively enriched gene sets: coagulation, lipids, metabolism, signaling pathways, cytoskeleton, and RNA/gene expression (NES ≥2, Nom *p* <0.05, FDR *q* <0.05; S3 Table). As seen with *P*. *gingivalis*, negatively enriched genes, including those involved in enzyme activity, signaling, and extracellular matrix, were affected but not significantly (NES >-2, FDR *q* >0.05; S4 Table).

**Fig 2:**
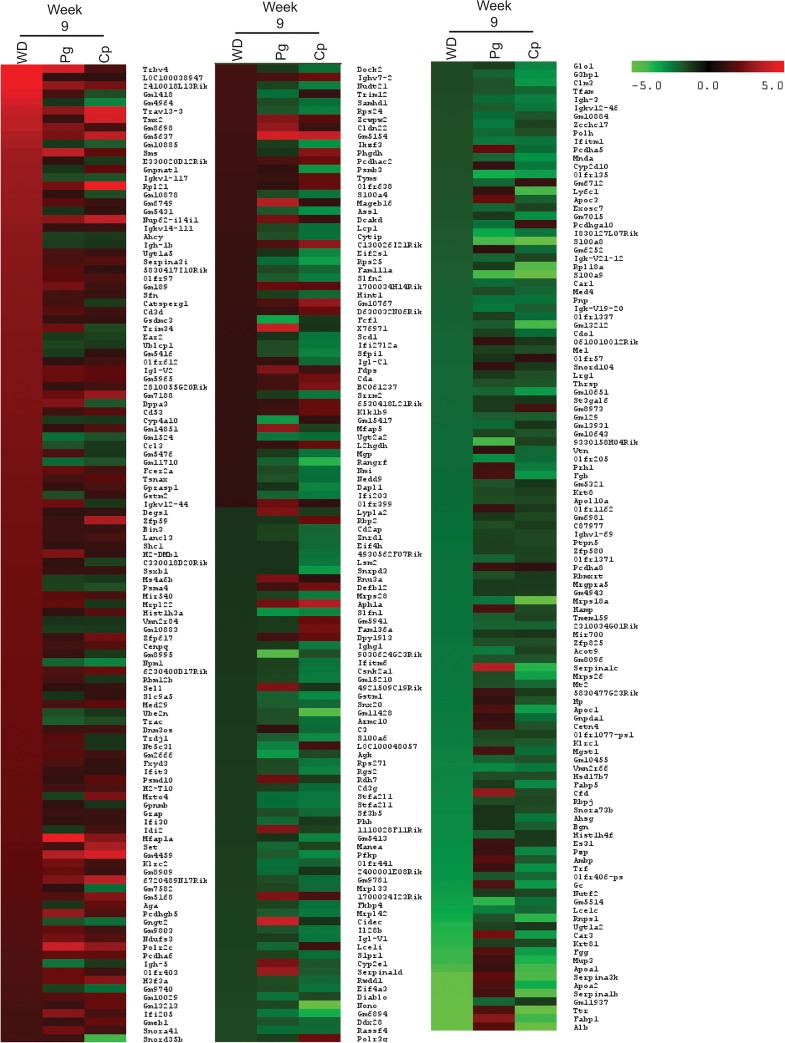
Genes upregulated and downregulated at week 9 in ApoE^-/-^ mice with bacterial infection or a Western diet. Heatmap shows the transcripts identified through microarray as upregulated or downregulated 2-fold or more by infection with *P*. *gingivalis* (Pg), *C*. *pneumoniae* (Cp), or fed a Western diet (WD) compared to Untreated Control in the ApoE^-/-^ Mice. Each condition represents RNA from 3 mice pooled.

Nine weeks following the last infection with *P*. *gingivalis*, *C*. *pneumoniae*, or 9 weeks of feeding a Western diet, ApoE^-/-^ mice were sacrificed in order to understand the changes in platelet transcripts before the formation of overt plaques. Following *P*. *gingivalis* infection, only 41 genes were increased and 32 were decreased (Fig 2). The top 5 genes that exhibited increased expression were MFAP1A (5.31-fold), TRBV4 (4.21-fold), POLR2C (3.99-fold), CIDEC (3.97-fold), and SMS (3.75-fold). Top 5 genes that exhibited decreased expression were S100A8 (-4.15-fold), S100A9 (-4.08-fold), OLFR135 (-3.45-fold), FCF1 (-3.26-fold), and AGK (-3.01-fold). GSEA identified numerous gene sets positively enriched, including coagulation and signaling pathways; however, these gene sets were not significantly altered by *P*. *gingivalis* infection (NES >-2, FDR *q* >0.05; S5 Table). It was the negatively enriched gene sets that were more significantly affected, including RNA/gene expression (NES ≤-2, Nom *p* <0.05, FDR *q* <0.05; S6 Table).

Nine weeks following the last infections with *C*.*pneumoniae*, there was differential effects on gene expression profiles as compared to that observed early after infection. Twenty-seven genes were increased and 109 decreased at week 9 compared to Untreated Control (Fig 2). The top 5 genes with increased expression at week 9 were RPL21 (5.52-fold), TRAV13-3 (4.34-fold), TMX2 (4.16-fold), NUP62-IL4I1 (3.81-fold), and APH1A (3.48-fold). The top 5 genes with decreased expression at week 9 were ALB (-16.46-fold), S100A9 (-9.38-fold), S100A8 (-9.38-fold), NONO (-6.79-fold), and TTR (-5.46-fold). Similarly to *P*. *gingivalis* at week 9, GSEA identified numerous gene sets positively enriched but that were not significantly affected by *C*. *pneumoniae* (S7 Table). Gene sets negatively enriched at week 9 were significant, including coagulation, RNA/gene expression, and inflammation (NES <-2, Nom *p* <0.01, FDR *q* <0.05; S8 Table).

**Fig 3:**
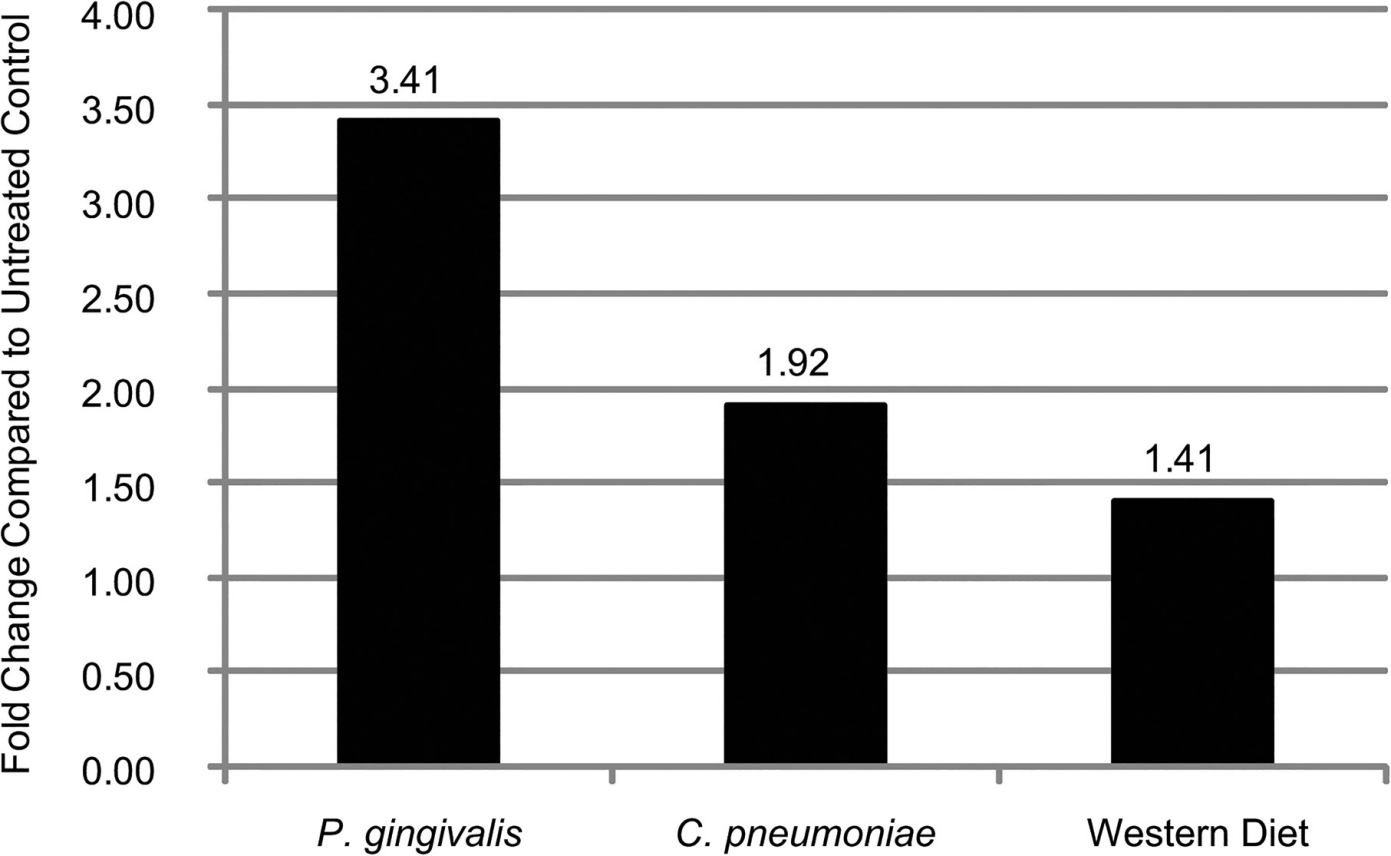
Verification of microarray analysis through qRT-PCR. Gene expression for MFAP1A at Week 9 identified through the microarray were verified using qRT-PCR. Each condition represents RNA from 3 mice pooled.

In mice fed a Western diet for 9 weeks, there were 67 genes increased and 67 genes decreased (Fig 2). The top 5 genes that exhibited increased expression as a result of a Western diet were TRBV4 (7.14-fold), TRAV13-3 (3.90-fold), TMX2 (3.74-fold), SMS (3.14-fold), and GNPNAT1 (3.05-fold). The top 5 genes with decreased expression with a Western diet were ALB (-18.34-fold), FABP1 (-6.89-fold), TTR (-5.51-fold), SERPINA1B (-4.95-fold), and APOA2 (-4.95-fold). Numerous gene sets were positively enriched, including those associated with DNA/proliferation, inflammation, RNA/gene expression, and signaling (NES >2, Nom *p* <0.05, FDR *q* <0.05; S9 Table). Only a few gene sets were negatively enriched, including those involved in coagulation and lipid (NES <-2, Nom *p* <0.01, FDR *q* <0.05; S10 Table). As a way to verify these results, qRT-PCR was run on week 9 samples for MFAP1A. As seen in Fig 3, there is an increase in its expression with *P*. *gingivalis* infection that is greater than the increase with *C*. *pneumoniae* infection or a Western diet, similar to the findings in the microarray.

### Differential Physiological Effects of Bacterial Infections or a Western Diet in ApoE^-/-^ Mice

**Fig 4:**
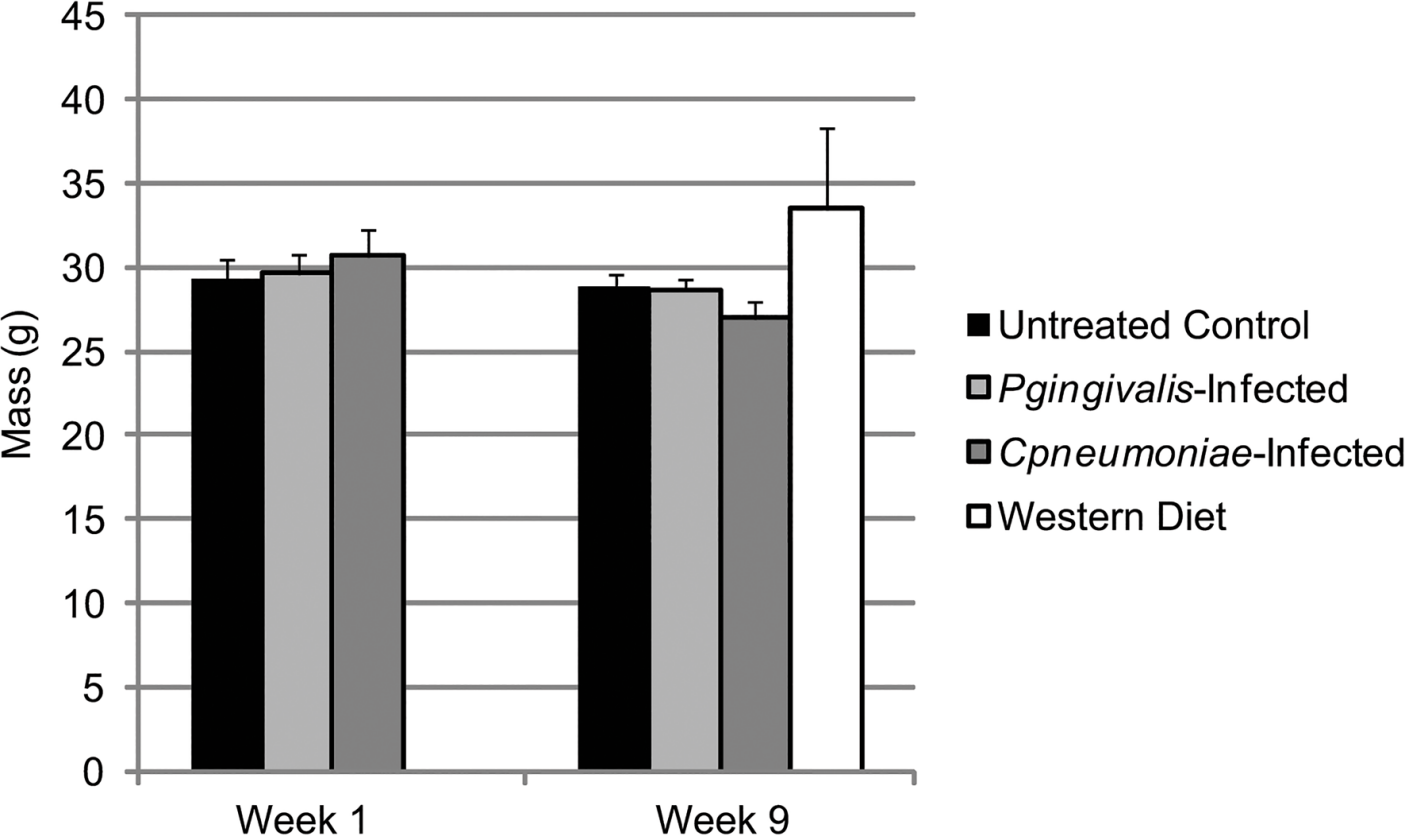
Change in ApoE^-/-^ mouse body mass with infection and diet. Mass was measured on Week 1, which was 24 h after the last *P*. *gingivalis* challenge and 4 days after *C*. *pneumoniae* exposure (n = 3 for each group), and on Week 9 (n = 3 for Untreated Control, *P*. *gingivalis*-Infected, and *C*. *pneumoniae*-Infected; n = 6 for Western Diet). Data is normally distributed and analyzed using an ANOVA.

**Fig 5:**
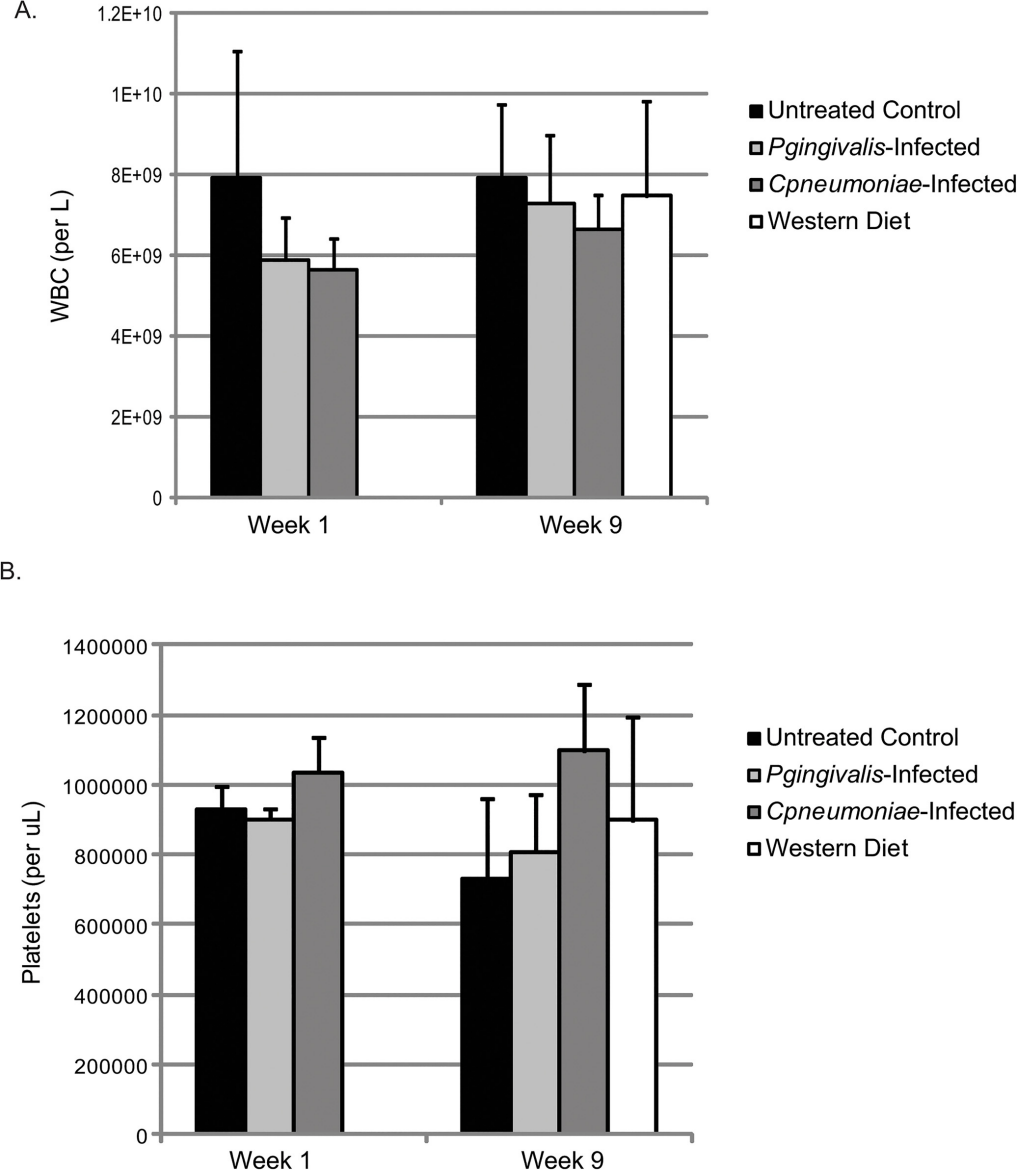
WBC and platelet count changes with infection and diet in the ApoE^-/-^ mice. Whole blood cell counts were assessed to determine the number of WBC and platelets. Measurements were performed on Week 1, which was 24 h after the last *P*. *gingivalis* challenge and 4 days after exposure to *C*. *pneumoniae* (n = 3 for each group), and on Week 9 (n = 3 for Untreated Control, *P*. *gingivalis*-Infected, and *C*. *pneumoniae*-Infected; n = 6 for Western Diet). Data is normally distributed and analyzed using an ANOVA.

**Fig 6:**
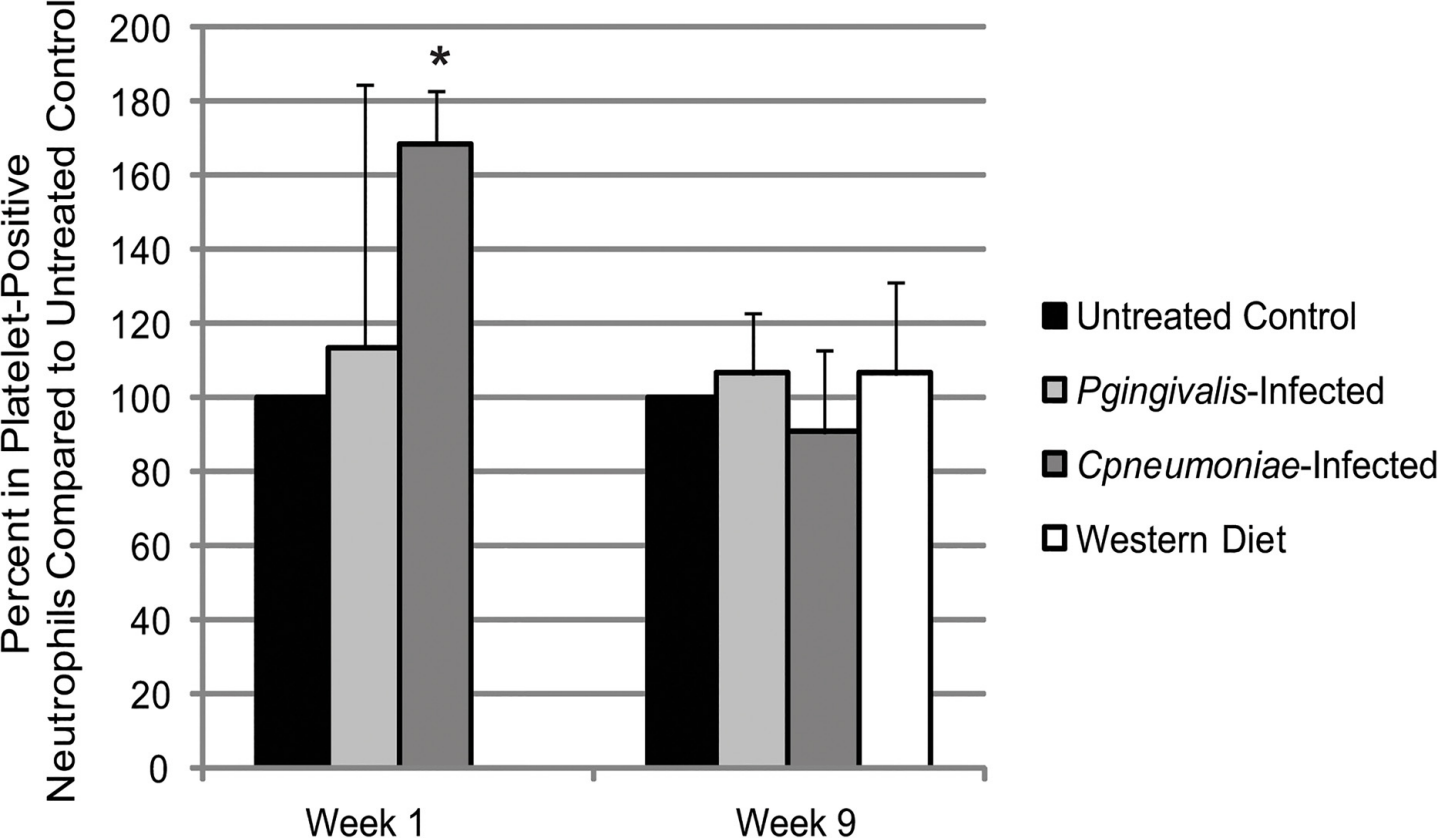
Heterotypic aggregate formation in ApoE^-/-^ mice with infection and diet. Whole blood samples taken on Week 1 (24 h after the last *P*. *gingivalis* challenge and 4 days after exposure to *C*. *pneumoniae*; n = 3 for each group) and on Week 9 (n = 3 for each group) and dual stained for platelet marker (CD41) and neutrophil marker (Ly6G). The percent of platelet-positive neutrophils were determined through flow cytometry and normalized to Untreated Control at each timepoint. Data is normally distributed and analyzed using an ANOVA. *p<0.05 compared to Untreated Control at Week 1.

**Fig 7:**
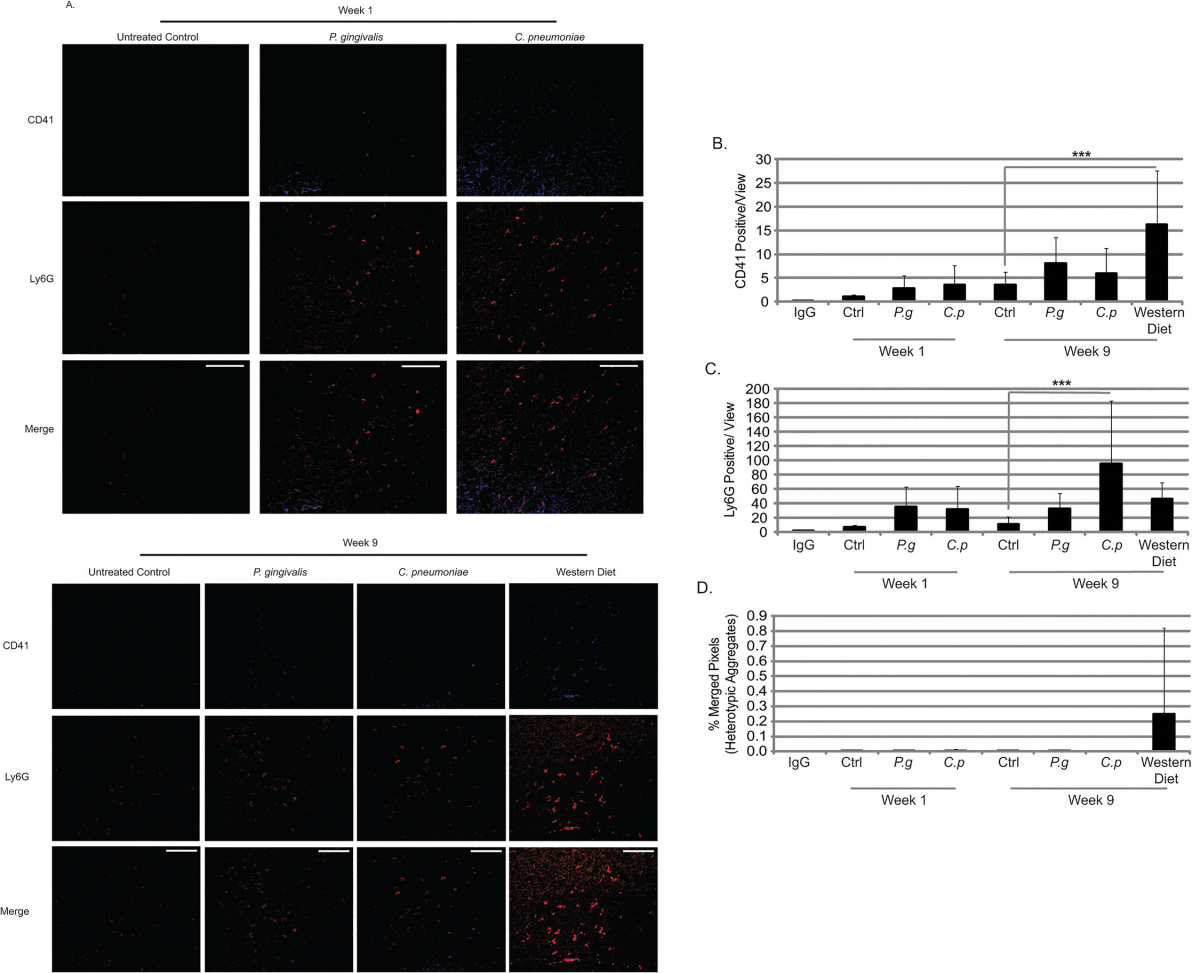
Platelet and neutrophil content in spleens in ApoE^-/-^ mice with infection and diet. (A) Spleen sections from ApoE^**-/-**^ mice at week 1 and week 9 were stained for CD41 (platelet marker; Alexa Fluor 405) and Ly6G (neutrophils; Texas Red) and visualized by confocal microscopy at 63X. The amount of CD41 staining (area; B), Ly6G staining (area; C), and the overlap of each signal (% pixels with overlapping signal); (D) were quantified. Data is normally distributed and analyzed using an ANOVA. n = 3 for each condition; ***p<0.001 compared to Untreated Control. Scale Bar in merged images = 50.0 μm.

There were no changes in body mass (Fig 4), or increases in white blood cell (WBC) counts (Fig 5A) at week 1 in ApoE^-/-^ mice infected with either *P*. *gingivalis* or *C*. *pneumoniae*. Only *C*. *pneumoniae* altered platelet counts (Fig 5B) compared to Untreated Control, although not significantly, suggesting this bacterial infection altered megakaryocyte maturation and platelet production and/or platelet clearance. Circulating heterotypic aggregates (platelet-neutrophil aggregates) were significantly increased in *C*. *pneumoniae* infected mice at week 1 (Fig 6); however, there were inconsistencies with the *P*. *gingivalis*-infected ApoE^-/-^ mice, but the data suggest that there was an increase in aggregate formation as well. Analysis of spleen sections from *P*. *gingivalis* or *C*. *pneumoniae* infected mice revealed increased CD41 levels, a platelet marker, and Ly6G levels, a neutrophil marker (Fig 7A–7C; IgG controls in S1 Fig). However, there were no heterotypic aggregates as indicated by the lack of overlap between the two signals when merged (Fig 7A and 7D).

At week 9, *P*. *gingivalis* or *C*. *pneumoniae* infections did not alter ApoE^-/-^ mouse body mass (Fig 4). However, in mice fed a Western diet, there was an increase in body mass by 5.2g. There was no effect on WBC counts at week 9 with either bacteria or a Western diet (Fig 5A). Platelet counts were still elevated following *C*. *pneumoniae* infection (Fig 5B). The increased circulating heterotypic aggregates seen with bacterial infections at week 1 were gone by week 9 (Fig 6). There were a greater number of platelets in the spleen sections from *P*. *gingivalis* or *C*. *pneumoniae* infected mice at week 9 as compared to week 1 (Fig 7A and 7B). *P*. *gingivalis* infection maintained the same amount of neutrophils in the spleen from week 1 to 9 (Fig 7A and 7C). For *C*. *pneumoniae*, there was a significantly greater number of neutrophils compared to Untreated Control at week 9 than that observed at week 1 (Fig 7A and 7C). A Western diet at week 9 did not increase circulating heterotypic aggregate formation. There was a significant increase in platelets in the spleens of mice fed a Western diet (Fig 7A and 7B) and a non-significant increase in neutrophils (Fig 7A and 7C). Unlike that observed following bacterial infections, there were heterotypic aggregates present in the spleens of mice fed a Western diet (Fig 7A and 7D).

### Changes in Platelet Transcripts Due to Diet in Participants of the Framingham Heart Study

To verify the relevance of the *in vivo* mouse observations, select platelet transcripts altered by a Western diet in the ApoE^-/-^ mice were measured in participants of the FHS (n = 1819; Table 1). These genes were either increased or decreased in the ApoE^-/-^ mice fed a Western diet, including albumin (ALB), α-1-microglobulin (AMBP), chemokine ligand 3 (CCL3), CD3 antigen (CD3D), CD53 antigen (CD53), fatty acid binding protein 1 (FABP1), fatty acid binding protein 5 (FABP5), fibrinogen γ chain (FGG), mitochondrial ribosomal protein S26 (MRPS26), nuclear receptor subfamily 2 (NR2F6), ribosomal protein L21 (RPL21), α-1-antitrypsin (SERPINA1), and vitronectin (VTN). These genes encompass various functional groups identified in the microarray, including coagulation, gene expression, inflammation, and lipids. These transcripts were compared to variables measured in the FHS that were associated with increased obesity [1,35] and a Western diet [26,36] in prior publications, including cholesterol, BMI, lipids, and triglyceride levels. A complete list of genes and their relationship with all studied clinical variables is found in S11 Table.

**Table 1:** Framingham heart study offspring cohort examination 8 characteristics.

Variables	Mean±Standard Deviation/Number (%)
Sample Size	1819
Women	993 (51)
Age (y)	67±9
BMI (kg/m^2^)	28.3±5.3
Systolic Blood Pressure (mm Hg)	129±17
Diastolic Blood Pressure (mm Hg)	73±10
Lipid Treatment	798 (44)
Total Cholesterol/HDL Ratio (mg/100 mL)	3.5±1.1
Triglycerides (mg/100 mL)	116±67
Glucose (mg/dL)	107±25
Antihypertensive Treatment	919 (51)
Aspirin (3 times/week)	823 (45)
Current Hormone Replacement Therapy	104 (6)
Diabetes mellitus	255 (14)
Prevalent Coronary Heart Disease	199 (11)
Smoker	155 (8.5)

Of the genes decreased in ApoE^-/-^ mice fed a Western diet, 4 out of the 7 (ALB, AMBP, FGG, and VTN) were also significantly decreased with a higher mean BMI or triglycerides in the FHS (Table 2 and S11 Table). FABP1 was not associated with cholesterol, BMI, or triglycerides, while FABP5 and SERPINA1 were increased with a higher mean in cholesterol or BMI. As for the genes that increased with a Western diet in the ApoE^-/-^ mice, 4 of the 5 (CD3D, CD53, NR2F6, and RPL21) were also increased significantly with a higher mean BMI, triglycerides, and/or cholesterol (Table 2 and S11 Table). CD53 and NR2F6 were increased significantly with an increased mean total cholesterol:HDL ratio and BMI but decreased significantly with increased mean triglyceride levels. One transcript, CCL3, although increased in the mice, was not increased in individuals in the FHS with these variables but was associated with other factors, such as diabetes (S11 Table).

**Table 2:** The relation between gene expression, clinical variables, and inflammatory markers in the FHS.

			FHS Fold Change (95% Conference Interval)				
Gene	Gene Function	Mouse Microarray Data (Fold Change Compared to UC)	Total Cholesterol: HDL^1^	Triglycerides^1^	BMI^2^	CRP^2^	IL6^2^
ALB	Fatty Acid Transport	0.055	—	0.90 (0.81, 0.99)*	—	—	—
FABP1	Fatty Acid Transport/ Metabolism	0.15	—	—	—	0.82 (0.74, 0.91)***	0.71 (0.60, 0.83)***
SERPINA1	Inflammation	0.20	—	—	1.13 (1.04, 1.23)**	1.29 (1.19, 1.40)***	1.50 (1.32, 1.72)***
FGG	Coagulation	0.26	—	—	0.87 (0.78, 0.97)*	0.83 (0.74, 0.92)***	0.68 (0.58, 0.80)***
AMBP	Coagulation	0.35	—	—	0.87 (0.78, 0.98)*	0.80 (0.72, 0.89)***	0.68 (0.57, 0.81)***
FABP5	Fatty Acid Transport/ Metabolism	0.39	1.15 (1.04, 1.26)**	—	—	1.10 (1.02, 1.18)*	1.12 (1.00, 1.26)*
MRPS26	Gene Expression- Mitoribosomes	0.43	—	—	—	1.14 (1.05, 1.22)***	1.22 (1.09, 1.38)***
VTN	Coagulation	0.49	—	0.87 (0.79, 0.96)**	—	—	0.85 (0.73, 0.99)*
CCL3	Inflammation	2.40	—	—	—	1.14 (1.05, 1.23)**	1.27 (1.13, 1.44)***
CD53	Inflammation	2.48	1.13 (1.02, 1.24)*	0.92 (0.85, 1.00)*	1.08 (1.00, 1.17)*	1.14 (1.05, 1.23)***	1.24 (1.10, 1.40)***
NR2F6	Gene Expression–Inflammation	2.55	1.15 (1.04, 1.27)**	0.92 (0.85, 1.00)*	1.11 (1.03, 1.20)**	1.17 (1.09, 1.26)***	1.26 (1.12, 1.42)***
CD3D	Inflammation	2.63	—	—	1.10 (1.01, 1.19)*	1.14 (1.05, 1.24)**	1.21 (1.07, 1.38)**
RPL21	Translation	3.00	—	—	1.11 (1.03, 1.19)**	1.12 (1.04, 1.20)**	1.21 (1.08, 1.35)**

^1^Fold Change for FHS: Total Cholesterol:HDL, BMI, Triglycerides – regression coefficient transformed (2^-β^) to express fold change in gene expression associated with 1 unit change in clinical covariate, with the exception in BMI (5 points) and triglycerides (50 points). *p<0.05, **p<0.01, ***p<0.001.

^2^ Fold Change for FHS: CRP and IL6 – regression coefficient transformed (2^-β^) to express fold change in gene expression associated with log_e_-CRP or log_e_-IL6 serum levels adjusted for multiple covariates. *p<0.05, **p<0.01, ***p<0.001.

Abbreviations: BMI – Body mass index, CRP – C-reactive protein, FHS – Framingham Heart Study, HDL – High density lipoprotein, IL6 – Interleukin 6, Total Cholesterol:HDL – Ratio between total and HDL cholesterol, UC – Untreated control.

Transcripts altered by a Western diet that were examined in FHS were also compared to circulating levels of CRP and IL6 (Table 2), inflammatory markers shown to be increased in individuals with CVD [37,38], obesity [35,39], and a Western diet [36,40,41]. As previously shown, these cytokines also associated significantly with inflammatory-related transcripts from platelets in FHS [2]. All but one transcript was significantly associated with higher mean levels of CRP and/or IL6. AMBP, FABP1, FGG, and VTN levels decreased with a higher mean level of CRP and/or IL6, which were all decreased in ApoE^-/-^ mice fed a Western diet and some in the FHS. CCL3, CD3D, CD53, FABP5, MRPS26, NR2F6, RPL21, and SERPINA1 were increased significantly with a higher mean CRP and/or IL6, which the majority were also increased in either FHS or in ApoE^-/-^ mice fed a Western diet. Only expression of ALB was not significantly associated with either of these cytokines.

## Discussion

The goal of the study was to characterize the effects of inflammatory stimuli–bacterial infections and a Western diet–on platelet transcripts and inflammatory function over time in a murine model of CVD. Similar effects on platelets were observed following infection with *P*. *gingivalis* and *C*. *pneumoniae* early after infection as determined by both transcriptional profiles and heterotypic aggregate formation. However, at a later timepoint (week 9), their effects on transcription diverged, with *C*. *pneumoniae* infection affecting platelets more similarly to a Western diet. The effects of a Western diet on platelet transcripts were confirmed in platelet samples from FHS.

Platelet transcripts and inflammatory function were measured at week 1 following either *P*. *gingivalis* or *C*. *pneumoniae* infection to establish the early responses to each bacterium in ApoE^-/-^ mice. Platelet transcripts were affected similarly by each bacterial infection, which suggests that each stimuli was activating through common pathways, most likely the TLR2-NF_К_B pathway, in the platelet precursor cell, the megakaryocyte, which we previously published on [42]. It is hypothesized that bacteria are recognized by megakaryocytes through their innate immune receptors. This hypothesis has been explored using Pam_3_CSK, a TLR2 synthetic agonist, in human and mouse megakaryocytes, showing upregulation of inflammatory and coagulation related transcripts [42]. Genes associated with RNA/gene expression and molecular transport, were also increased in platelets following infection with *P*. *gingivalis* and *C*. *penumoniae*. It is hypothesized the increase in gene expression correlates to an increase in mRNA translation within the platelets, as has been seen with platelets in response to cytokines [2,43]. Additionally, the increased number of transcripts in both of these groups could be transferred to other vascular cells, such as endothelial cells or monocytes [44], to increase translation in these cells in response to infection. We hypothesized transcriptional changes would be reflective with functional modifications in the platelets. As seen at week 1, there was an increase in circulating heterotypic aggregates and positive enrichment of inflammatory gene sets (*i*.*e*., complement cascade). Not only is platelet production affected but so is the platelet response. As previously shown, platelets respond to Pam_3_CSK4, *P*. *gingivalis* [3,4], and *C*. *pneumoniae* (data not shown) through TLR2 and IL1R1. This results in platelets binding to neutrophils to form heterotypic aggregates, as well as increased P-selectin surface expression, and adhesion to collagen, increasing the risk of thrombosis as seen in human studies [22,45].

**Fig 8:**
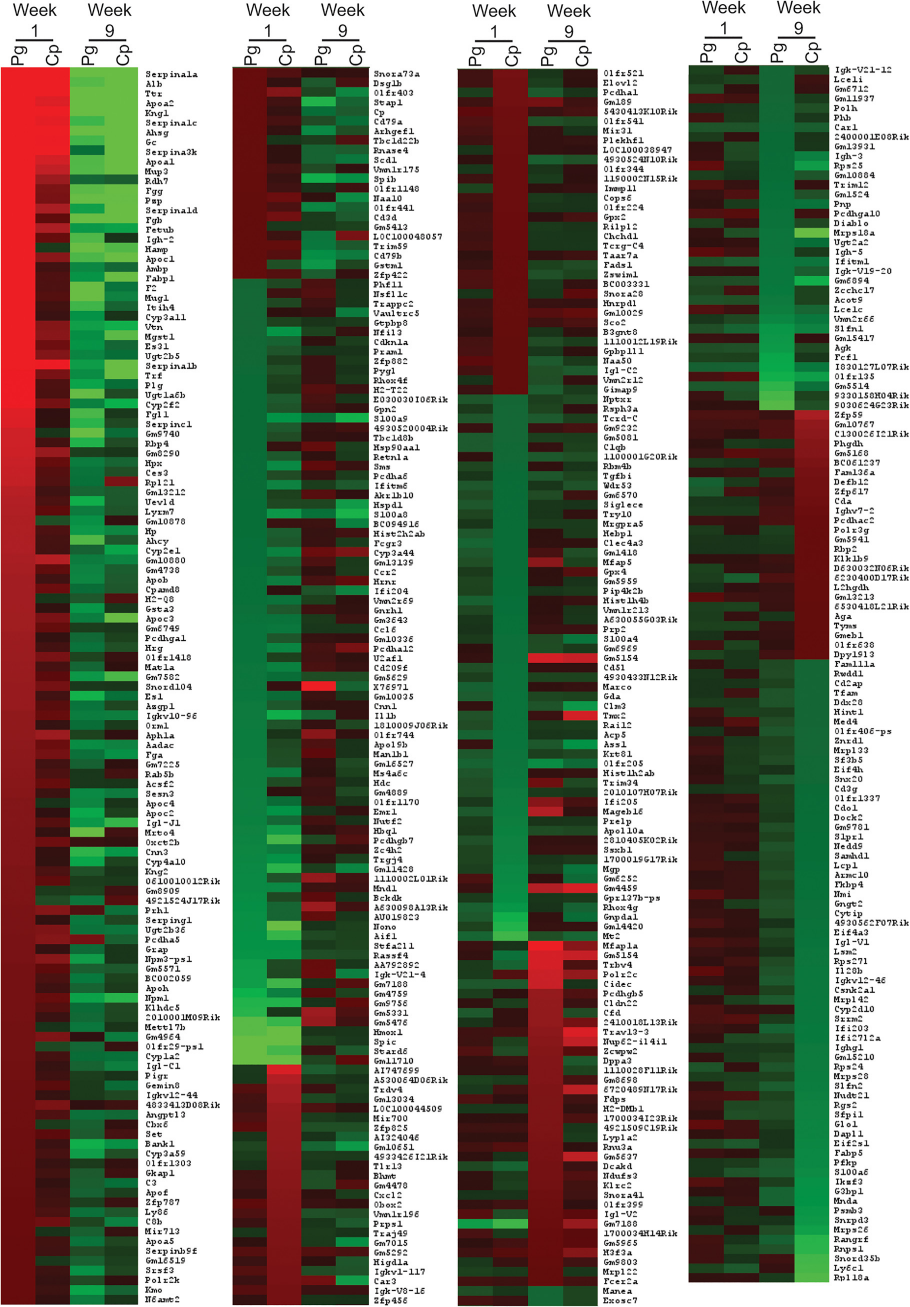
Comparison of genes affected by both bacterial infections at week 1 and week 9. Heatmap shows the transcripts identified through microarray as upregulated or downregulated 2-fold or more with either *P*. *gingivalis* (Pg) or *C*. *pneumoniae* (Cp) infection compared to Untreated Control in the ApoE^**-/-**^ mice at week 1 and 9. Each condition represents RNA from 3 mice pooled.

At week 9, following *P*. *gingivalis* and *C*. *pneumoniae* infections, before overt plaque formation had yet to occur in the aorta, alterations in both platelet transcripts and inflammatory function are still occurring. Although not directly measured in our study, previous work by our group and others have shown that each bacterium is still present in the systems of these mice. An increase in circulating *P*. *gingivalis*-specific IgG has been detected through 13 weeks post infection [46] and by 24 weeks, this bacterium is identified in aorta, heart, and liver tissue [47]. As for *C*. *pneumoniae*, there is a measurable serum IgG level up to 16 weeks, with bacteria detected in the lungs, aorta, heart, and spleen [48]. Overall, both bacteria upregulated fewer genes at the later timepoint compared that observed at week 1 (Fig 8). Infection with *C*. *pneumoniae* resulted in more downregulation in gene expression, which was further supported by GSEA, showing significant negative enrichment in gene sets. Unlike with *C*. *pneumoniae*, *P*. *gingivalis* infection alterations in gene transcript changes were diminished at week 9. It is hypothesized that over the course of the infection, megakaryocytes are slowly returning to baseline in terms of the types of RNA produced, with fewer genes and gene groups being altered. Further, changes in transcripts are also reflective of platelet function. Platelet-neutrophil aggregates are no longer in circulation at week 9. The cells were individually present in the spleens obtained from these mice. It is possible that we no longer see these cells as aggregates even in the spleens because the interaction between the platelets and neutrophils is transient, a brief interaction that results in activation of either/both cells and transfer of information on the current environment. With more platelets in the spleen, it is expected that there would be a decrease in circulating platelet levels. Instead, the platelet concentrations stay steady if not increase. This data suggest megakaryocytes are still responding to the infection, directly or indirectly.

With a Western diet, it was expected that lipid associated genes would be upregulated in platelets, but instead, as seen in FHS [1,2,3], there was an increase in inflammatory related transcripts, as well as signaling and proliferation. As previously seen with the FHS [1], the changes associated with a Western diet on platelets can either be due to the diet, change in body weight, or the proinflammatory setting. Further work is needed to understand this relationship. Interestingly, many genes and/or gene function groups altered by a Western diet were also altered following a *C*. *pneumoniae* infection. Genes downregulated following a Western diet or *C*. *pneumoniae* infection included TTR, GC, ALB, APOA2, and FABP1. Functionally, the Western diet did not produce circulating heterotypic aggregates but did promote the formation of aggregates to the vessel wall, as seen in the spleens. It is hypothesized that a Western diet, promotes platelets adhesion to the blood vessels, which promotes adhesion of immune cells as well, leading to the formation of atherosclerosis [49].

The effects of a Western diet in the ApoE^-/-^ mice were verified using platelet transcripts from the FHS. Thirteen genes were identified from the microarray data, including ALB, AMBP, CCL3, CD3D, CD53, FABP1, FABP5, FGG, MRPS26, NR2F6, RPL21, SERPINA1, and VTN. Twelve out of the 13 genes had altered gene expression that correlated to one or more clinical variables associated with adiposity, including BMI, triglycerides, and cholesterol. Additionally, inflammatory related cytokines, CRP and IL6, which are increased with CVD and associate significantly with inflammatory related transcripts from platelets in FHS [2], were associated with changes in 12 of the 13 identified transcripts, again, supporting the notion that platelets react with an inflammatory stimuli resembles their reaction to a Western diet.

In conjunction with the analysis of platelets in these ApoE^-/-^ mice, aortic tissue, consisting of endothelial cells, immune cells, and smooth muscle cells, were also examined by microarray. At week 1, *P*. *gingivalis* infection had little effect on gene expression in the aortic tissue. *C*. *pneumoniae* infection upregulated genes associated with GPCR signaling, viral myocarditis, antigen processing/presentation, and membranes in the entire aortic tissue [23], which is in contrast to what is shown here in platelets. When comparing the platelet data to the results from the aortic tissue at week 9, we see there are no gene sets similarly affected with either bacterial infection [23]. In fact, two pathways positively enriched in aortic tissue, PPAR signaling and peptide chain formation, are negatively enriched in platelets. As for a Western diet, there were only 4 similarities found in the positively enriched gene sets, with consisted of B cell receptor signaling pathways, host interactions of HIV factors, cell cycle checkpoints, and M-G1 transition.

### Strengths and Limitations

Our study has substantial advantages including a translational breadth with experimental and epidemiological data relating transcripts from mouse and human platelets and mouse platelet function, human lipid, adiposity, and inflammatory data from FHS. However, we note the following caveats in our study. Our bacterial and Western diet exposure data were conducted in 3 mice, so we may have been underpowered to detect modest effect sizes. We could not verify any confounding attributions due to antibiotic pre-treatment with the *P*. *gingivalis* group for all genes identified; however, we were able to show in a couple of genes they were not altered by antibiotics (sham treatment) compared to Untreated Control in C57BL/6J mice (S2 Fig). Additionally, there are discrepancies between human and mouse data due to species differences [50]. Our FHS data were observational and cross-sectional–we cannot rule out residual confounding or establish the temporality or causal nature of the associations we report. In addition, our cohort was constituted of largely middle-aged to older adults of European ancestry; we cannot determine the generalizability of our findings to younger individuals or other races/ethnicities. Finally, it should be noted that we did not correct for the number of statistical comparisons made when examining the relationships between each gene and clinical covariates in the FHS samples. To reduce the probability of type I error, we focused interpretation on only those covariates of most relevance given the mouse experiments examining a Western diet.

## Conclusion

Overall, as has been seen with other immune cells, platelets respond differentially to distinct inflammatory etiologies. Platelets early response to an infection with an oral bacterium or a respiratory bacterium, both associated with CVD, were similar; however, over time, the effects on platelets by each bacterium diverge. Interestingly, *C*. *pneumoniae* alters platelet transcripts similarly to a Western diet at week 9. In conclusion, the inflammatory-induced transcriptional and functional changes measured in platelets appear to reflect systemic changes occurring with disease development, and provides insight into progression and regression.

## Supporting Information

S1 Fig IgG staining of spleen sections from ApoE^-/-^ mice. (EPS)

S2 Fig Effects of antibiotics (sham) on gene expression in C57BL/6J. (EPS)

S1 Table Positively enriched gene sets in platelets from ApoE-/- mice infected with *P. gingivalis* compared to untreated control–at week 1. (DOCX)

S2 Table Negatively enriched gene sets in platelets from ApoE-/- mice infected with *P. gingivalis* compared to untreated control–at week 1. (DOCX)

S3 Table Positively enriched gene sets in platelets from ApoE-/- mice infected with *C. pneumoniae* compared to untreated control–at week 1. (DOCX)

S4 Table Negatively enriched gene sets in platelets from ApoE-/- mice infected with *C. pneumoniae* compared to untreated control–at week 1. (DOCX)

S5 Table Positively enriched gene sets in platelets from ApoE-/- mice infected with *P. gingivalis* compared to untreated control–at week 9. (DOCX)

S6 Table Negatively enriched gene sets in platelets from ApoE-/- mice infected with *P. gingivalis* compared to untreated control–at week 9. (DOCX)

S7 Table Positively enriched gene sets in platelets from ApoE-/- mice infected with *C. pneumoniae* compared to untreated control–at week 9. (DOCX)

S8 Table Negatively enriched gene sets in platelets from ApoE^-/-^ mice infected with *C*. *pneumoniae* compared to untreated control–at week 9. (DOCX)

S9 Table Positively enriched gene sets in platelets from ApoE-/- mice on a Western diet compared to untreated control–at week 9. (DOCX)

S10 Table Negatively enriched gene sets in platelets from ApoE-/- mice on a Western diet compared to untreated control–at week 9. (DOCX)

S11 Table Relations between gene expression and clinical variable in FHS. (DOCX)
