# Supplementary material for: Specific Inflammatory Stimuli Lead to Distinct Platelet Responses in Mice and Humans
Source: PLoS One. 2015 Jul 6;10(7):e0131688. doi: 10.1371/journal.pone.0131688 (PMC4493099; doi:10.1371/journal.pone.0131688)
Supplement: S1 Table — (DOCX) [file pone.0131688.s003.docx]

| **S1 Table: Positively Enriched Gene Sets in Platelets From ApoE^-/-^ Mice Infected with *P. gingivalis* Compared to Untreated Control – at Week 1.** | | | | | |
| --- | --- | --- | --- | --- | --- |
| **NAME** | **SIZE** | **ES** | **NES** | **NOM**  ***p*-val** | **FDR**  ***q*-val** |
| PLATELET DEGRANULATION | 80 | 0.731 | 2.302 | 0.000 | 0.000 |
| LIPOPROTEIN METABOLISM | 26 | 0.888 | 2.272 | 0.000 | 0.000 |
| INTRINSIC PATHWAY | 23 | 0.903 | 2.249 | 0.000 | 0.000 |
| COMPLEMENT AND COAGULATION CASCADES | 62 | 0.744 | 2.232 | 0.000 | 0.000 |
| REGULATION OF GENE EXPRESSION IN BETA CELLS | 56 | 0.732 | 2.149 | 0.000 | 0.000 |
| PEPTIDE CHAIN ELONGATION | 38 | 0.763 | 2.093 | 0.000 | 0.000 |
| FORMATION OF FIBRIN CLOT CLOTTING CASCADE | 31 | 0.798 | 2.104 | 0.000 | 0.000 |
| REGULATION OF BETA CELL DEVELOPMENT | 69 | 0.683 | 2.070 | 0.000 | 0.000 |
| CHYLOMICRON MEDIATED LIPID TRANSPORT | 17 | 0.885 | 2.104 | 0.000 | 0.000 |
| GENE EXPRESSION | 357 | 0.553 | 2.074 | 0.000 | 0.000 |
| RIBOSOME | 39 | 0.743 | 2.079 | 0.000 | 0.000 |
| AMI PATHWAY | 19 | 0.890 | 2.109 | 0.000 | 0.000 |
| INFLUENZA LIFE CYCLE | 87 | 0.659 | 2.080 | 0.000 | 0.000 |
| INFLUENZA VIRAL RNA TRANSCRIPTION AND REPLICATION | 54 | 0.703 | 2.086 | 0.000 | 0.001 |
| VIRAL MRNA TRANSLATION | 39 | 0.757 | 2.114 | 0.000 | 0.001 |
| FORMATION OF A POOL OF FREE 40S SUBUNITS | 49 | 0.737 | 2.114 | 0.000 | 0.001 |
| RNA BINDING | 195 | 0.580 | 2.051 | 0.000 | 0.001 |
| STRUCTURAL CONSTITUENT OF RIBOSOME | 44 | 0.726 | 2.033 | 0.000 | 0.001 |
| LIPID TRANSPORT | 26 | 0.788 | 2.023 | 0.000 | 0.001 |
| REACTOME GTP HYDROLYSIS AND JOINING OF THE 60S RIBOSOMAL SUBUNIT | 58 | 0.679 | 1.995 | 0.000 | 0.002 |
| HIV1 TRANSCRIPTION ELONGATION | 37 | 0.713 | 1.991 | 0.000 | 0.002 |
| PLATELET ACTIVATION | 158 | 0.571 | 1.978 | 0.000 | 0.003 |
| PLATELET AGGREGATION PLUG FORMATION | 26 | 0.779 | 1.981 | 0.000 | 0.003 |
| PROTEIN HETERODIMERIZATION ACTIVITY | 71 | 0.639 | 1.982 | 0.000 | 0.003 |
| GRB2 SOS LINKAGE TO MAPK SIGNALING TO INTERGRINS | 15 | 0.884 | 1.976 | 0.000 | 0.003 |
| TRANSLATION | 71 | 0.643 | 1.983 | 0.000 | 0.003 |
| NEGATIVE REGULATION OF MULTICELLULAR ORGANISMAL PROCESS | 28 | 0.741 | 1.959 | 0.000 | 0.003 |
| INTEGRIN αIIB β3 SIGNALING | 23 | 0.794 | 1.957 | 0.000 | 0.003 |
| EXTRACELLULAR SPACE | 209 | 0.545 | 1.938 | 0.000 | 0.004 |
| FORMATION OF PLATELET PLUG | 177 | 0.556 | 1.936 | 0.000 | 0.004 |
| P130CAS LINKAGE TO MAPK SIGNALING FOR INTEGRINS | 15 | 0.851 | 1.930 | 0.000 | 0.005 |
| TRANSLATION INITIATION COMPLEX FORMATION | 38 | 0.689 | 1.927 | 0.002 | 0.005 |
| REGULATION OF PROTEIN STABILITY | 18 | 0.811 | 1.924 | 0.000 | 0.005 |
| TAT MEDIATED HIV1 ELONGATION ARREST, RECOVERY | 27 | 0.739 | 1.901 | 0.000 | 0.007 |
| RIBONUCLEOPROTEIN COMPLEX BIOGENESIS, ASSEMBLY | 71 | 0.628 | 1.903 | 0.000 | 0.007 |
| CYTOKINE SECRETION | 15 | 0.848 | 1.905 | 0.000 | 0.007 |
| NUCLEOLUS | 105 | 0.572 | 1.893 | 0.000 | 0.007 |
| INTEGRIN CELL SURFACE INTERACTIONS | 80 | 0.606 | 1.896 | 0.000 | 0.007 |
| FORMATION OF TERNARY COMPLEX AND 43S COMPLEX | 32 | 0.720 | 1.893 | 0.000 | 0.007 |
| INSULIN SYNTHESIS AND SECRETION | 81 | 0.600 | 1.882 | 0.000 | 0.009 |
| TRANSCRIPTION OF THE HIV GENOME | 55 | 0.639 | 1.881 | 0.000 | 0.009 |
| OXYGEN BINDING | 19 | 0.799 | 1.874 | 0.000 | 0.010 |
| PEPTIDYL TYROSINE MODIFICATION | 27 | 0.723 | 1.875 | 0.000 | 0.010 |
| FORMATION AND MATURATION OF mRNA TRANSCRIPT | 137 | 0.551 | 1.868 | 0.000 | 0.011 |
| IMMUNE EFFECTOR PROCESS | 33 | 0.700 | 1.859 | 0.002 | 0.012 |
| RNA POLII CTD PHOSPHORYLATION AND INTERACTION WITH CE | 24 | 0.744 | 1.854 | 0.002 | 0.013 |
| HIV LIFE CYCLE | 94 | 0.573 | 1.850 | 0.000 | 0.013 |
| LATE PHASE OF HIV LIFE CYCLE | 82 | 0.597 | 1.851 | 0.000 | 0.013 |
| LIPID TRANSPORTER ACTIVITY | 23 | 0.729 | 1.847 | 0.000 | 0.013 |
| ELONGATION, PROCESSING OF CAPPED TRANSCRIPTS | 119 | 0.552 | 1.845 | 0.000 | 0.014 |
| HEMOSTASIS | 262 | 0.505 | 1.839 | 0.000 | 0.014 |
| PEPTIDYL TYROSINE PHOSPHORYLATION | 25 | 0.724 | 1.831 | 0.000 | 0.016 |
| ATP DEPENDENT HELICASE ACTIVITY | 24 | 0.736 | 1.826 | 0.002 | 0.017 |
| PPAR SIGNALING PATHWAY | 67 | 0.596 | 1.819 | 0.000 | 0.018 |
| mRNA PROCESSING | 30 | 0.695 | 1.816 | 0.002 | 0.019 |
| RNA POLYMERASE II TRANSCRIPTION | 84 | 0.569 | 1.808 | 0.002 | 0.020 |
| REGULATION OF HYDROLASE ACTIVITY | 65 | 0.599 | 1.810 | 0.000 | 0.020 |
| ANTIGEN BINDING | 16 | 0.766 | 1.787 | 0.004 | 0.027 |
| METABOLISM OF XENOBIOTICS BY CYTOCHROME P450 | 52 | 0.615 | 1.783 | 0.000 | 0.028 |
| PHASE II CONJUGATION | 44 | 0.617 | 1.772 | 0.004 | 0.031 |
| LIPID BINDING | 73 | 0.586 | 1.773 | 0.000 | 0.032 |
| FURTHER PLATELET RELEASATE | 19 | 0.756 | 1.774 | 0.000 | 0.032 |
| tRNA AMINOACYLATION | 40 | 0.631 | 1.766 | 0.002 | 0.034 |
| STEROID BINDING | 17 | 0.755 | 1.754 | 0.006 | 0.036 |
| FORMATION OF THE EARLY ELONGATION COMPLEX | 29 | 0.673 | 1.758 | 0.004 | 0.036 |
| INTRINSIC PATHWAY | 17 | 0.745 | 1.759 | 0.002 | 0.036 |
| PROTEIN RNA COMPLEX ASSEMBLY | 56 | 0.604 | 1.757 | 0.002 | 0.036 |
| RHO PROTEIN SIGNAL TRANSDUCTION | 34 | 0.645 | 1.760 | 0.000 | 0.036 |
| REGULATION OF CELL MIGRATION | 26 | 0.693 | 1.754 | 0.006 | 0.036 |
| MAINTENANCE OF LOCALIZATION | 19 | 0.733 | 1.752 | 0.004 | 0.036 |
| RNA POLYMERASE I TRANSCRIPTION TERMINATION | 19 | 0.732 | 1.754 | 0.006 | 0.037 |
| miRNA BIOGENESIS | 17 | 0.750 | 1.748 | 0.010 | 0.038 |
| PD1 SIGNALING | 18 | 0.740 | 1.744 | 0.002 | 0.039 |
| PPARα PATHWAY | 56 | 0.583 | 1.737 | 0.000 | 0.042 |
| IMMUNE RESPONSE | 205 | 0.486 | 1.733 | 0.000 | 0.042 |
| PROCESSING OF CAPPED INTRON CONTAINING PRE MRNA | 123 | 0.521 | 1.736 | 0.000 | 0.043 |
| mRNA SPLICING | 95 | 0.549 | 1.733 | 0.000 | 0.043 |
| REGULATION OF SECRETION | 36 | 0.631 | 1.733 | 0.004 | 0.043 |
| REGULATION OF PROTEIN SECRETION | 18 | 0.724 | 1.721 | 0.006 | 0.048 |
| DOWNSTREAM TCR SIGNALING | 36 | 0.638 | 1.722 | 0.005 | 0.048 |
| EXTRACELLULAR REGION PART | 297 | 0.471 | 1.718 | 0.000 | 0.048 |
| RNA PROCESSING | 146 | 0.502 | 1.716 | 0.000 | 0.049 |
| MEDIATOR COMPLEX | 18 | 0.730 | 1.713 | 0.004 | 0.050 |

SIZE – Number of genes; ES – Enrichment Score; NES – Normalized Enrichement Score; NOM *p*-val – Nominal *p*-value; FDR *q*-val – False Discovery Rate.
