# Supplementary material for: Specific Inflammatory Stimuli Lead to Distinct Platelet Responses in Mice and Humans
Source: PLoS One. 2015 Jul 6;10(7):e0131688. doi: 10.1371/journal.pone.0131688 (PMC4493099; doi:10.1371/journal.pone.0131688)
Supplement: S2 Table — (DOCX) [file pone.0131688.s004.docx]

| **S2 Table: Negatively Enriched Gene Sets in Platelets From ApoE^-/-^ Mice Infected with *P. gingivalis***  **Compared to Untreated Control – at Week 1.** | | | | |  |  |
| --- | --- | --- | --- | --- | --- | --- |
| **NAME** | **SIZE** | **ES** | **NES** | | **NOM *p*-val** | **FDR *q*-val** |
| METALLOPEPTIDASE ACTIVITY | 49 | -0.505 | -1.541 | | 0.016 | 0.834 |
| ACTIVATION OF NMDA RECEPTOR UPON GLUTAMATE BINDING AND POSTSYNAPTIC EVENTS | 33 | -0.547 | -1.547 | | 0.020 | 0.860 |
| β-ALANINE METABOLISM | 22 | -0.613 | -1.553 | | 0.029 | 0.897 |
| TRAFFICKING OF AMPA RECEPTORS | 29 | -0.555 | -1.503 | | 0.029 | 0.979 |
| CONTRACTILE FIBER | 21 | -0.591 | -1.512 | | 0.052 | 0.982 |
| MUSCLE DEVELOPMENT | 90 | -0.434 | -1.493 | | 0.007 | 0.994 |
| EXTRACELLULAR MATRIX STRUCTURAL CONSTITUENT | 24 | -0.608 | -1.553 | | 0.029 | 0.996 |
| THIOLESTER HYDROLASE ACTIVITY | 16 | -0.676 | -1.612 | | 0.022 | 1.000 |
| MEIOTIC CELL CYCLE | 33 | -0.567 | -1.593 | | 0.020 | 1.000 |
| LYSOSOME | 116 | -0.453 | -1.589 | | 0.002 | 1.000 |
| OTHER GLYCAN DEGRADATION | 16 | -0.681 | -1.577 | | 0.022 | 1.000 |
| EXOPEPTIDASE ACTIVITY | 30 | -0.578 | -1.563 | | 0.014 | 1.000 |
| PROPANOATE METABOLISM | 29 | -0.588 | -1.559 | | 0.025 | 1.000 |
| SMOOTH MUSCLE CONTRACTION | 17 | -0.638 | -1.558 | | 0.025 | 1.000 |
| CALCIUM ION BINDING | 88 | -0.460 | -1.555 | | 0.002 | 1.000 |
| MUSCLE CELL DIFFERENTIATION | 21 | -0.585 | -1.477 | | 0.049 | 1.000 |
| SYNAPTIC VESICLE | 15 | -0.634 | -1.457 | | 0.054 | 1.000 |
| AMINO SUGAR AND NUCLEOTIDE SUGAR METABOLISM | 43 | -0.489 | -1.452 | | 0.034 | 1.000 |
| GLYCOPROTEIN METABOLIC PROCESS | 81 | -0.435 | -1.451 | | 0.013 | 1.000 |
| MEIOSIS I | 20 | -0.574 | -1.450 | | 0.056 | 1.000 |

SIZE – Number of genes; ES – Enrichment Score; NES – Normalized Enrichement Score; NOM *p*-val – Nominal *p*-value; FDR *q*-val – False Discovery Rate.
