# Supplementary material for: Specific Inflammatory Stimuli Lead to Distinct Platelet Responses in Mice and Humans
Source: PLoS One. 2015 Jul 6;10(7):e0131688. doi: 10.1371/journal.pone.0131688 (PMC4493099; doi:10.1371/journal.pone.0131688)
Supplement: S3 Table — (DOCX) [file pone.0131688.s005.docx]

| **S3 Table: Positively Enriched Gene Sets in Platelets From ApoE^-/-^ Mice Infected with *C. pneumoniae* Compared to Untreated Control – at Week 1.** | | | | | |
| --- | --- | --- | --- | --- | --- |
| **NAME** | **SIZE** | **ES** | **NES** | **NOM *p*-val** | **FDR *q*-val** |
| LIPOPROTEIN METABOLISM | 26 | 0.775 | 2.083 | 0.000 | 0.008 |
| LIPID TRANSPORT | 26 | 0.738 | 1.986 | 0.000 | 0.015 |
| PLATELET DEGRANULATION | 80 | 0.597 | 1.994 | 0.000 | 0.017 |
| AMI PATHWAY | 19 | 0.780 | 1.924 | 0.000 | 0.026 |
| ORGANELLE LOCALIZATION | 24 | 0.731 | 1.893 | 0.002 | 0.027 |
| INTRINSIC PATHWAY | 23 | 0.731 | 1.909 | 0.000 | 0.030 |
| MAINTENANCE OF LOCALIZATION | 19 | 0.758 | 1.895 | 0.000 | 0.030 |
| COMPLEMENT AND COAGULATION CASCADES | 62 | 0.610 | 1.925 | 0.000 | 0.032 |
| CHYLOMICRON MEDIATED LIPID TRANSPORT | 17 | 0.769 | 1.898 | 0.002 | 0.032 |
| PROTEIN HETERODIMERIZATION ACTIVITY | 71 | 0.569 | 1.871 | 0.000 | 0.034 |
| TRANSCRIPTION OF THE HIV GENOME | 55 | 0.594 | 1.855 | 0.000 | 0.040 |
| P130CAS LINKAGE TO MAPK SIGNALING FOR INTEGRINS | 15 | 0.777 | 1.837 | 0.002 | 0.043 |
| RESPONSE TO NUTRIENT LEVELS | 28 | 0.688 | 1.839 | 0.000 | 0.046 |
| STEROID BINDING | 17 | 0.744 | 1.802 | 0.000 | 0.065 |
| HELICASE ACTIVITY | 46 | 0.595 | 1.779 | 0.000 | 0.065 |
| REGULATION OF PROTEIN STABILITY | 18 | 0.732 | 1.794 | 0.000 | 0.067 |
| GRB2 SOS PROVIDES LINKAGE TO MAPK SIGNALING FOR INTERGRINS | 15 | 0.771 | 1.788 | 0.002 | 0.067 |
| CYTOKINE SECRETION | 15 | 0.762 | 1.779 | 0.004 | 0.068 |
| FORMATION AND MATURATION OF MRNA TRANSCRIPT | 137 | 0.493 | 1.781 | 0.000 | 0.070 |
| SELENOAMINO ACID METABOLISM | 26 | 0.662 | 1.766 | 0.006 | 0.075 |

SIZE – Number of genes; ES – Enrichment Score; NES – Normalized Enrichement Score; NOM *p*-val – Nominal *p*-value; FDR *q*-val – False Discovery Rate.
