# Supplementary material for: Specific Inflammatory Stimuli Lead to Distinct Platelet Responses in Mice and Humans
Source: PLoS One. 2015 Jul 6;10(7):e0131688. doi: 10.1371/journal.pone.0131688 (PMC4493099; doi:10.1371/journal.pone.0131688)
Supplement: S4 Table — (DOCX) [file pone.0131688.s006.docx]

| **S4 Table: Negatively Enriched Gene Sets in Platelets From ApoE^-/-^ Mice Infected with *C. pneumoniae* Compared to Untreated Control – at Week 1.** | | | | | |
| --- | --- | --- | --- | --- | --- |
| **NAME** | **SIZE** | **ES** | **NES** | **NOM *p*-val** | **FDR *q*-val** |
| CALCIUM ION BINDING | 88 | -0.535 | -1.789 | 0.000 | 0.256 |
| MITOCHONDRIAL RIBOSOME | 20 | -0.699 | -1.742 | 0.002 | 0.260 |
| IL1R PATHWAY | 32 | -0.658 | -1.834 | 0.000 | 0.266 |
| EXTRACELLULAR MATRIX STRUCTURAL CONSTITUENT | 24 | -0.709 | -1.803 | 0.000 | 0.282 |
| ORGANELLAR RIBOSOME | 20 | -0.699 | -1.745 | 0.008 | 0.290 |
| TOLL PATHWAY | 36 | -0.616 | -1.758 | 0.000 | 0.296 |
| GSK3 PATHWAY | 27 | -0.636 | -1.689 | 0.004 | 0.422 |
| GLYCOSAMINOGLYCAN DEGRADATION | 21 | -0.742 | -1.848 | 0.000 | 0.424 |
| RIBOSOMAL SUBUNIT | 18 | -0.690 | -1.679 | 0.008 | 0.426 |
| RIBOSOME | 30 | -0.598 | -1.651 | 0.008 | 0.468 |
| ACETYLCHOLINE BINDING | 17 | -0.688 | -1.631 | 0.013 | 0.475 |
| LYSOSOME | 116 | -0.465 | -1.614 | 0.004 | 0.486 |
| LEISHMANIA INFECTION | 61 | -0.512 | -1.637 | 0.002 | 0.487 |
| NTHI PATHWAY | 23 | -0.640 | -1.654 | 0.008 | 0.498 |
| NEGATIVE REGULATION OF CELL DIFFERENTIATION | 25 | -0.611 | -1.602 | 0.022 | 0.505 |
| REGULATION OF CELL DIFFERENTIATION | 55 | -0.524 | -1.617 | 0.004 | 0.508 |
| REGULATION OF MYELOID CELL DIFFERENTIATION | 16 | -0.670 | -1.586 | 0.015 | 0.555 |
| DIGESTION | 36 | -0.566 | -1.579 | 0.017 | 0.556 |
| CATION BINDING | 188 | -0.423 | -1.560 | 0.000 | 0.624 |
| NFκB PATHWAY | 23 | -0.604 | -1.549 | 0.023 | 0.653 |

SIZE – Number of genes; ES – Enrichment Score; NES – Normalized Enrichement Score; NOM *p*-val – Nominal *p*-value; FDR *q*-val – False Discovery Rate.
