# Supplementary material for: Specific Inflammatory Stimuli Lead to Distinct Platelet Responses in Mice and Humans
Source: PLoS One. 2015 Jul 6;10(7):e0131688. doi: 10.1371/journal.pone.0131688 (PMC4493099; doi:10.1371/journal.pone.0131688)
Supplement: S5 Table — (DOCX) [file pone.0131688.s007.docx]

| **S5 Table: Positively Enriched Gene Sets in Platelets From ApoE^-/-^ Mice Infected with *P. gingivalis* Compared to Untreated Control – at Week 9.** | | | | | |
| --- | --- | --- | --- | --- | --- |
| **NAME** | **SIZE** | **ES** | **NES** | **NOM *p*-val** | **FDR *q*-val** |
| NEUROPEPTIDE RECEPTOR ACTIVITY | 21 | 0.750 | 1.912 | 0.002 | 0.052 |
| COMPLEMENT AND COAGULATION CASCADES | 62 | 0.623 | 1.942 | 0.000 | 0.060 |
| NEUROPEPTIDE BINDING | 22 | 0.721 | 1.821 | 0.002 | 0.093 |
| PEPTIDE RECEPTOR ACTIVITY | 48 | 0.605 | 1.802 | 0.000 | 0.100 |
| GLUCONEOGENESIS | 28 | 0.685 | 1.822 | 0.002 | 0.116 |
| PLATELET DEGRANULATION | 80 | 0.560 | 1.836 | 0.000 | 0.128 |
| COMPLEMENT CASCADE | 18 | 0.727 | 1.752 | 0.018 | 0.172 |
| RESPONSE TO NUTRIENT | 16 | 0.738 | 1.738 | 0.009 | 0.180 |
| AMI PATHWAY | 19 | 0.702 | 1.709 | 0.007 | 0.227 |
| RESPONSE TO EXTRACELLULAR STIMULUS | 32 | 0.585 | 1.636 | 0.011 | 0.248 |
| COMP PATHWAY | 16 | 0.708 | 1.666 | 0.006 | 0.252 |
| G-PROTEIN COUPLED RECEPTOR ACTIVITY | 172 | 0.448 | 1.651 | 0.000 | 0.255 |
| METABOLISM OF BILE ACIDS AND BILE SALTS | 26 | 0.613 | 1.638 | 0.016 | 0.256 |
| RESPONSE TO NUTRIENT LEVELS | 28 | 0.622 | 1.658 | 0.009 | 0.257 |
| PROTEIN SECRETION | 28 | 0.620 | 1.678 | 0.004 | 0.262 |
| INTRINSIC PATHWAY | 23 | 0.648 | 1.669 | 0.007 | 0.265 |
| PEPTIDE BINDING | 79 | 0.500 | 1.639 | 0.002 | 0.268 |
| RHODOPSIN LIKE RECEPTOR ACTIVITY | 123 | 0.480 | 1.682 | 0.000 | 0.276 |
| DETECTION OF CHEMICAL STIMULUS | 15 | 0.706 | 1.608 | 0.027 | 0.309 |
| STRUCTURAL CONSTITUENT OF MUSCLE | 26 | 0.603 | 1.598 | 0.015 | 0.324 |

SIZE – Number of genes; ES – Enrichment Score; NES – Normalized Enrichement Score; NOM *p*-val – Nominal *p*-value; FDR *q*-val – False Discovery Rate.
