# Supplementary material for: Specific Inflammatory Stimuli Lead to Distinct Platelet Responses in Mice and Humans
Source: PLoS One. 2015 Jul 6;10(7):e0131688. doi: 10.1371/journal.pone.0131688 (PMC4493099; doi:10.1371/journal.pone.0131688)
Supplement: S6 Table — (DOCX) [file pone.0131688.s008.docx]

| **S6 Table: Negatively Enriched Gene Set in Platelets From ApoE^-/-^ Mice Infected with *P. gingivalis* Compared to Untreated Control – at Week 9.** | | | | | |
| --- | --- | --- | --- | --- | --- |
| **NAME** | **SIZE** | **ES** | **NES** | **NOM *p*-val** | **FDR *q*-val** |
| TRANSPORT OF MATURE mRNA DERIVED FROM AN INTRON CONTAINING TRANSCRIPT | 45 | -0.641 | -1.847 | 0.000 | 0.036 |
| TRANSPORT OF THE SLBP INDEPENDENT MATURE mRNA | 29 | -0.701 | -1.840 | 0.000 | 0.036 |
| SPLICEOSOME | 108 | -0.579 | -1.911 | 0.000 | 0.038 |
| FORMATION OF THE TERNARY COMPLEX, 43S COMPLEX | 32 | -0.709 | -1.887 | 0.000 | 0.038 |
| FORMATION OF A POOL OF FREE 40S SUBUNITS | 49 | -0.641 | -1.831 | 0.000 | 0.038 |
| ATP DEPENDENT RNA HELICASE ACTIVITY | 15 | -0.807 | -1.854 | 0.000 | 0.039 |
| TRANSLATION INITIATION COMPLEX FORMATION | 38 | -0.669 | -1.872 | 0.000 | 0.042 |
| GTP HYDROLYSIS, JOINING OF 60S RIBOSOMAL SUBUNIT | 58 | -0.626 | -1.859 | 0.000 | 0.043 |
| RNA HELICASE ACTIVITY | 22 | -0.776 | -1.926 | 0.000 | 0.055 |
| RNA DEPENDENT ATPASE ACTIVITY | 16 | -0.779 | -1.789 | 0.002 | 0.060 |
| ATP DEPENDENT HELICASE ACTIVITY | 24 | -0.705 | -1.793 | 0.002 | 0.061 |
| ACTIVATION OF NFκB TRANSCRIPTION FACTOR | 18 | -0.752 | -1.798 | 0.004 | 0.062 |
| SPLICEOSOME | 38 | -0.642 | -1.782 | 0.002 | 0.062 |
| RNA SPLICING | 76 | -0.572 | -1.776 | 0.000 | 0.064 |
| GENE EXPRESSION | 357 | -0.468 | -1.759 | 0.000 | 0.067 |
| RNA BINDING | 195 | -0.495 | -1.765 | 0.000 | 0.070 |
| POSITIVE REGULATION OF TRANSCRIPTION FACTOR ACTIVITY | 24 | -0.694 | -1.759 | 0.006 | 0.070 |
| tRNA METABOLIC PROCESS | 18 | -0.735 | -1.738 | 0.004 | 0.080 |
| ANTIGEN PROCESSING AND PRESENTATION | 49 | -0.604 | -1.741 | 0.000 | 0.081 |
| POSITIVE REGULATION OF BINDING | 28 | -0.655 | -1.734 | 0.002 | 0.081 |

SIZE – Number of genes; ES – Enrichment Score; NES – Normalized Enrichement Score; NOM *p*-val – Nominal *p*-value; FDR *q*-val – False Discovery Rate.
