# Supplementary material for: Specific Inflammatory Stimuli Lead to Distinct Platelet Responses in Mice and Humans
Source: PLoS One. 2015 Jul 6;10(7):e0131688. doi: 10.1371/journal.pone.0131688 (PMC4493099; doi:10.1371/journal.pone.0131688)
Supplement: S7 Table — (DOCX) [file pone.0131688.s009.docx]

| **S7 Table: Positively Enriched Gene Sets in Platelets From ApoE^-/-^ Mice Infected with *C. pneumoniae* Compared to Untreated Control – at Week 9.** | | | | | |
| --- | --- | --- | --- | --- | --- |
| **NAME** | **SIZE** | **ES** | **NES** | **NOM *p*-val** | **FDR *q*-val** |
| NEUROTRANSMITTER RECEPTOR ACTIVITY | 49 | 0.658 | 2.007 | 0.000 | 0.017 |
| NEUROPEPTIDE RECEPTOR ACTIVITY | 21 | 0.771 | 1.966 | 0.000 | 0.018 |
| NEUROTRANSMITTER BINDING | 52 | 0.604 | 1.848 | 0.003 | 0.058 |
| NEUROPEPTIDE BINDING | 22 | 0.737 | 1.849 | 0.000 | 0.073 |
| APICAL PART OF CELL | 17 | 0.764 | 1.850 | 0.000 | 0.094 |
| PEPTIDE RECEPTOR ACTIVITY | 48 | 0.587 | 1.771 | 0.000 | 0.139 |
| ACETYLCHOLINE BINDING | 17 | 0.738 | 1.737 | 0.000 | 0.160 |
| ANION CATION SYMPORTER ACTIVITY | 15 | 0.737 | 1.745 | 0.009 | 0.164 |
| RESPONSE TO NUTRIENT | 16 | 0.725 | 1.711 | 0.012 | 0.197 |
| PHOSPHOLIPASE C ACTIVITY | 15 | 0.719 | 1.675 | 0.010 | 0.261 |
| STRUCTURAL CONSTITUENT OF MUSCLE | 26 | 0.617 | 1.642 | 0.005 | 0.306 |
| SYNAPTIC VESICLE | 15 | 0.701 | 1.648 | 0.017 | 0.311 |
| NEGATIVE REGULATION OF DNA METABOLIC PROCESS | 16 | 0.685 | 1.611 | 0.020 | 0.385 |
| SECRETORY GRANULE | 17 | 0.648 | 1.578 | 0.032 | 0.424 |
| SYMPORTER ACTIVITY | 30 | 0.576 | 1.590 | 0.010 | 0.432 |
| RHODOPSIN LIKE RECEPTOR ACTIVITY | 123 | 0.445 | 1.581 | 0.004 | 0.440 |
| TRANS GOLGI NETWORK | 18 | 0.647 | 1.541 | 0.030 | 0.464 |
| SECONDARY ACTIVE TRANSMEMBRANE TRANSPORTER ACTIVITY | 44 | 0.509 | 1.544 | 0.019 | 0.477 |
| LYSOSOME | 116 | 0.437 | 1.545 | 0.007 | 0.500 |
| REGULATION OF PEPTIDYL TYROSINE PHOSPHORYLATION | 16 | 0.653 | 1.547 | 0.031 | 0.520 |

SIZE – Number of genes; ES – Enrichment Score; NES – Normalized Enrichement Score; NOM *p*-val – Nominal *p*-value; FDR *q*-val – False Discovery Rate.
