# Supplementary material for: Specific Inflammatory Stimuli Lead to Distinct Platelet Responses in Mice and Humans
Source: PLoS One. 2015 Jul 6;10(7):e0131688. doi: 10.1371/journal.pone.0131688 (PMC4493099; doi:10.1371/journal.pone.0131688)
Supplement: S8 Table — (DOCX) [file pone.0131688.s010.docx]

| **S8 Table: Negatively Enriched Gene Sets in Platelets From ApoE^-/-^ Mice Infected with *C. pneumoniae* Compared to Untreated Control – at Week 9.** | | | | | |
| --- | --- | --- | --- | --- | --- |
| **NAME** | **SIZE** | **ES** | **NES** | **NOM *p*-val** | **FDR *q*-val** |
| TRANSLATION | 71 | -0.753 | -2.207 | 0.000 | 0.000 |
| GTP HYDROLYSIS, JOINING OF 60S RIBOSOMAL SUBUNIT | 58 | -0.780 | -2.194 | 0.000 | 0.000 |
| PEPTIDE CHAIN ELONGATION | 38 | -0.819 | -2.169 | 0.000 | 0.000 |
| FORMATION OF A POOL OF FREE 40S SUBUNITS | 49 | -0.798 | -2.155 | 0.000 | 0.000 |
| VIRAL MRNA TRANSLATION | 39 | -0.801 | -2.135 | 0.000 | 0.000 |
| RIBOSOME | 39 | -0.807 | -2.115 | 0.000 | 0.000 |
| REGULATION OF GENE EXPRESSION IN BETA CELLS | 56 | -0.741 | -2.095 | 0.000 | 0.000 |
| SPLICEOSOME | 108 | -0.660 | -2.065 | 0.000 | 0.000 |
| STRUCTURAL CONSTITUENT OF RIBOSOME | 44 | -0.758 | -2.042 | 0.000 | 0.000 |
| TRANSLATION INITIATION COMPLEX FORMATION | 38 | -0.770 | -2.052 | 0.000 | 0.000 |
| RNA SPLICING | 76 | -0.689 | -2.021 | 0.000 | 0.000 |
| REGULATION OF BETA CELL DEVELOPMENT | 69 | -0.700 | -2.033 | 0.000 | 0.000 |
| PLATELET DEGRANULATION | 80 | -0.675 | -1.997 | 0.000 | 0.001 |
| FORMATION OF THE TERNARY COMPLEX, 43S COMPLEX | 32 | -0.787 | -1.998 | 0.000 | 0.001 |
| RNA BINDING | 195 | -0.600 | -1.986 | 0.000 | 0.001 |
| RNA HELICASE ACTIVITY | 22 | -0.836 | -1.982 | 0.000 | 0.001 |
| ANTIGEN PROCESSING AND PRESENTATION | 49 | -0.724 | -1.986 | 0.000 | 0.001 |
| INFLUENZA LIFE CYCLE | 87 | -0.652 | -1.967 | 0.000 | 0.001 |
| ATP DEPENDENT HELICASE ACTIVITY | 24 | -0.811 | -1.968 | 0.000 | 0.001 |
| REGULATION OF LIPID METABOLISM BY PPARα | 60 | -0.688 | -1.954 | 0.000 | 0.001 |
| GENE EXPRESSION | 357 | -0.563 | -1.955 | 0.000 | 0.001 |
| PPAR SIGNALING PATHWAY | 67 | -0.679 | -1.956 | 0.000 | 0.001 |
| INFLUENZA VIRAL RNA TRANSCRIPTION, REPLICATION | 54 | -0.689 | -1.958 | 0.000 | 0.001 |
| RNA PROCESSING | 146 | -0.607 | -1.944 | 0.000 | 0.001 |
| LIPOPROTEIN METABOLISM | 26 | -0.783 | -1.937 | 0.000 | 0.002 |
| PPARα PATHWAY | 56 | -0.680 | -1.928 | 0.000 | 0.002 |

SIZE – Number of genes; ES – Enrichment Score; NES – Normalized Enrichement Score; NOM *p*-val – Nominal *p*-value; FDR *q*-val – False Discovery Rate.
