# Supplementary material for: Specific Inflammatory Stimuli Lead to Distinct Platelet Responses in Mice and Humans
Source: PLoS One. 2015 Jul 6;10(7):e0131688. doi: 10.1371/journal.pone.0131688 (PMC4493099; doi:10.1371/journal.pone.0131688)
Supplement: S9 Table — (DOCX) [file pone.0131688.s011.docx]

| **S9 Table: Positively Enriched Gene Sets in Platelets From ApoE^-/-^ Mice on a Western Diet Compared to Untreated Control – at Week 9.** | | | | | |
| --- | --- | --- | --- | --- | --- |
| **NAME** | **SIZE** | **ES** | **NES** | **NOM *p*-val** | **FDR *q*-val** |
| M-G1 TRANSITION | 59 | 0.678 | 2.125 | 0.000 | 0.000 |
| ORC1 REMOVAL FROM CHROMATIN | 61 | 0.665 | 2.084 | 0.000 | 0.001 |
| CDT1 ASSOCIATION WITH THE CDC6 ORC ORIGIN COMPLEX | 50 | 0.687 | 2.072 | 0.000 | 0.001 |
| P53-INDEPENDENT DNA DAMAGE RESPONSE | 41 | 0.727 | 2.096 | 0.000 | 0.001 |
| HOST INTERACTIONS OF HIV FACTORS | 108 | 0.590 | 2.033 | 0.000 | 0.001 |
| DNA REPLICATION PRE INITIATION | 73 | 0.631 | 2.041 | 0.000 | 0.001 |
| PD1 SIGNALING | 18 | 0.843 | 2.045 | 0.000 | 0.001 |
| STABILIZATION OF P53 | 44 | 0.715 | 2.056 | 0.000 | 0.001 |
| VIF MEDIATED DEGRADATION OF APOBEC3G | 43 | 0.718 | 2.096 | 0.000 | 0.002 |
| SIGNALING BY WNT | 56 | 0.639 | 1.995 | 0.000 | 0.002 |
| DOWNSTREAM TCR SIGNALING | 36 | 0.711 | 1.979 | 0.000 | 0.003 |
| REGULATION OF ORNITHINE DECARBOXYLASE | 46 | 0.667 | 1.961 | 0.000 | 0.003 |
| PROTEASOME | 42 | 0.675 | 1.965 | 0.000 | 0.003 |
| AUTODEGRADATION OF CDH1 BY CDH1 APC | 54 | 0.642 | 1.963 | 0.000 | 0.003 |
| SYNTHESIS OF DNA | 86 | 0.594 | 1.966 | 0.000 | 0.004 |
| SCF β TRCP MEDIATED DEGRADATION OF EMI1 | 46 | 0.660 | 1.927 | 0.000 | 0.005 |
| TRANSLATION INITIATION COMPLEX FORMATION | 38 | 0.660 | 1.909 | 0.002 | 0.006 |
| TCR SIGNALING | 52 | 0.627 | 1.911 | 0.000 | 0.007 |
| CDC20 PHOSPHO-APC MEDIATED DEGRADATION OF CYCLIN A | 60 | 0.613 | 1.897 | 0.000 | 0.009 |
| FORMATION OF THE TERNARY COMPLEX, 43S COMPLEX | 32 | 0.686 | 1.875 | 0.000 | 0.012 |
| PHOSPHORYLATION OF CD3 AND TCR ζ CHAINS | 15 | 0.803 | 1.869 | 0.000 | 0.013 |
| REGULATION OF APC ACTIVATORS BETWEEN G1-S, EARLY ANAPHASE | 67 | 0.580 | 1.857 | 0.000 | 0.015 |
| T CELL ACTIVATION | 42 | 0.633 | 1.846 | 0.002 | 0.017 |
| SCF SKP2 MEDIATED DEGRADATION OF P27 P21 | 50 | 0.615 | 1.847 | 0.000 | 0.017 |
| PREFOLDIN MEDIATED TRANSFER OF SUBSTRATE TO CCT TRIC | 25 | 0.686 | 1.842 | 0.000 | 0.018 |
| GENERATION OF SECOND MESSENGER MOLECULES | 25 | 0.715 | 1.833 | 0.000 | 0.019 |
| DNA DAMAGE RESPONSE SIGNAL TRANSDUCTION | 33 | 0.665 | 1.821 | 0.000 | 0.021 |
| TRANSLATION | 71 | 0.567 | 1.816 | 0.000 | 0.022 |
| ASTHMA | 21 | 0.706 | 1.807 | 0.002 | 0.024 |
| GTPASE REGULATOR ACTIVITY | 55 | 0.583 | 1.802 | 0.000 | 0.024 |
| REGULATION OF ACTION POTENTIAL | 16 | 0.768 | 1.808 | 0.004 | 0.024 |
| REACTOME S PHASE | 100 | 0.532 | 1.804 | 0.000 | 0.024 |
| MITOSIS | 80 | 0.546 | 1.797 | 0.000 | 0.025 |
| CELL CYCLE CHECKPOINTS | 105 | 0.530 | 1.788 | 0.000 | 0.027 |
| RAS GUANYL NUCLEOTIDE EXCHANGE FACTOR ACTIVITY | 17 | 0.749 | 1.768 | 0.006 | 0.032 |
| M PHASE OF MITOTIC CELL CYCLE | 82 | 0.540 | 1.766 | 0.000 | 0.033 |
| HEMATOPOIETIC CELL LINEAGE | 73 | 0.553 | 1.772 | 0.000 | 0.033 |
| CYCLIN E ASSOCIATED EVENTS DURING G1-S TRANSITION | 56 | 0.577 | 1.768 | 0.002 | 0.033 |
| HIV INFECTION | 168 | 0.496 | 1.770 | 0.000 | 0.033 |
| INFLUENZA VIRAL RNA TRANSCRIPTION AND REPLICATION | 54 | 0.570 | 1.757 | 0.000 | 0.036 |
| MITOTIC M M-G1 PHASES | 151 | 0.494 | 1.754 | 0.000 | 0.037 |
| PROTEIN MODIFICATION BY SMALL PROTEIN CONJUGATION | 41 | 0.596 | 1.750 | 0.002 | 0.038 |
| B CELL RECEPTOR SIGNALING PATHWAY | 73 | 0.534 | 1.739 | 0.000 | 0.041 |
| CHAPERONIN MEDIATED PROTEIN FOLDING | 46 | 0.575 | 1.741 | 0.000 | 0.042 |
| UBIQUITIN CYCLE | 46 | 0.595 | 1.739 | 0.000 | 0.042 |
| METABOLISM OF PROTEINS | 162 | 0.482 | 1.735 | 0.000 | 0.042 |
| CYSTEINE TYPE ENDOPEPTIDASE ACTIVITY | 37 | 0.615 | 1.730 | 0.000 | 0.045 |
| M PHASE | 109 | 0.510 | 1.727 | 0.000 | 0.046 |

SIZE – Number of genes; ES – Enrichment Score; NES – Normalized Enrichement Score; NOM *p*-val – Nominal *p*-value; FDR *q*-val – False Discovery Rate.
