# Supplementary material for: Specific Inflammatory Stimuli Lead to Distinct Platelet Responses in Mice and Humans
Source: PLoS One. 2015 Jul 6;10(7):e0131688. doi: 10.1371/journal.pone.0131688 (PMC4493099; doi:10.1371/journal.pone.0131688)
Supplement: S10 Table — (DOCX) [file pone.0131688.s012.docx]

| **S10 Table: Negatively Enriched Gene Set in Platelets From ApoE^-/-^ Mice on a Western Diet Compared to Untreated Control – at Week 9.** | | | | | |
| --- | --- | --- | --- | --- | --- |
| **NAME** | **SIZE** | **ES** | **NES** | **NOM *p*-val** | **FDR *q*-val** |
| PLATELET DEGRANULATION | 80 | -0.665 | -2.147 | 0.000 | 0.001 |
| LIPOPROTEIN METABOLISM | 26 | -0.749 | -1.971 | 0.000 | 0.021 |
| OXYGEN BINDING | 19 | -0.776 | -1.951 | 0.000 | 0.022 |
| P130 CAS LINKAGE TO MAPK SIGNALING FOR INTEGRINS | 15 | -0.778 | -1.791 | 0.004 | 0.069 |
| CYTOKINE SECRETION | 15 | -0.768 | -1.774 | 0.006 | 0.074 |
| REGULATION OF PROTEIN SECRETION | 18 | -0.733 | -1.791 | 0.002 | 0.075 |
| AMI PATHWAY | 19 | -0.748 | -1.797 | 0.000 | 0.076 |
| PPAR SIGNALING PATHWAY | 67 | -0.591 | -1.860 | 0.000 | 0.080 |
| PPAR$\alpha$ PATHWAY | 56 | -0.601 | -1.844 | 0.000 | 0.080 |
| IMMUNE EFFECTOR PROCESS | 33 | -0.664 | -1.820 | 0.000 | 0.080 |
| ANTIGEN BINDING | 16 | -0.771 | -1.774 | 0.004 | 0.080 |
| REGULATION OF PROTEIN STABILITY | 18 | -0.749 | -1.800 | 0.002 | 0.082 |
| COMPLEMENT AND COAGULATION CASCADES | 62 | -0.581 | -1.807 | 0.002 | 0.085 |
| LIPID TRANSPORT | 26 | -0.695 | -1.824 | 0.000 | 0.089 |
| GAP JUNCTION ASSEMBLY | 17 | -0.747 | -1.744 | 0.004 | 0.100 |
| PROTEIN SECRETION | 28 | -0.643 | -1.732 | 0.000 | 0.109 |
| COLLAGEN | 23 | -0.676 | -1.720 | 0.000 | 0.112 |
| METABOLISM OF BILE ACIDS AND BILE SALTS | 26 | -0.641 | -1.721 | 0.006 | 0.117 |
| REGULATION OF MAP3K CASCADE | 17 | -0.718 | -1.709 | 0.010 | 0.120 |
| REGULATION OF HEART CONTRACTION | 22 | -0.664 | -1.704 | 0.008 | 0.121 |

SIZE – Number of genes; ES – Enrichment Score; NES – Normalized Enrichement Score; NOM *p*-val – Nominal *p*-value; FDR *q*-val – False Discovery Rate.
