# Supplementary material for: Specific Inflammatory Stimuli Lead to Distinct Platelet Responses in Mice and Humans
Source: PLoS One. 2015 Jul 6;10(7):e0131688. doi: 10.1371/journal.pone.0131688 (PMC4493099; doi:10.1371/journal.pone.0131688)
Supplement: S11 Table — (DOCX) [file pone.0131688.s013.docx]

**S11 Table: Relations Between Gene Expression and Clinical Variables in FHS.**

| **Gene Symbol** | **FHS Clinical Variable** | **Fold Change^1^** | ***p*-value** | **Regression Coefficient** | **Lower 95% CI** | **Upper 95% CI** |
| --- | --- | --- | --- | --- | --- | --- |
| ALB | Men | 1.355884433 | 0.01 | -0.439234158 | -0.751835269 | -0.126633047 |
| ALB | Triglycerides | 0.895260155 | 0.03 | 0.003192422 | 0.000311626 | 0.006073218 |
| ALB | Aspirin 3X/Week | 1.276171565 | 0.03 | -0.351822318 | -0.663639528 | -0.040005109 |
| AMBP | Men | 0.660523891 | 0.001 | 0.598317335 | 0.25383669 | 0.94279798 |
| AMBP | Antilipid R_x_ | 0.674799085 | 0.002 | 0.567470104 | 0.202499378 | 0.93244083 |
| AMBP | BMI | 0.873817921 | 0.02 | 0.038919084 | 0.00652732 | 0.071310848 |
| AMBP | Aspirin 3X/Week | 1.317548633 | 0.02 | -0.397856194 | -0.741472994 | -0.054239395 |
| CCL3 | Age | 1.181825876 | 0.003 | -0.024101755 | -0.040239869 | -0.007963641 |
| CCL3 | Men | 1.244618535 | 0.01 | -0.315703592 | -0.561465329 | -0.069941856 |
| CCL3 | Antihypertensive R_x_ | 1.230527163 | 0.02 | -0.299276523 | -0.556862836 | -0.04169021 |
| CCL3 | Diabetes | 1.317789912 | 0.03 | -0.398120377 | -0.747841568 | -0.048399186 |
| CD3D | Men | 1.939570785 | 0.0005 | -0.955737416 | -1.21202884 | -0.699445992 |
| CD3D | BMI | 1.097084761 | 0.03 | -0.026735 | -0.050834268 | -0.002635732 |
| CD3D | Diabetes | 1.307060242 | 0.04 | -0.386325649 | -0.751030681 | -0.021620616 |
| CD53 | Men | 1.74677074 | 0.0005 | -0.804690282 | -1.047312907 | -0.562067658 |
| CD53 | Total Cholesterol:HDL Ratio | 1.126685858 | 0.02 | -0.172085288 | -0.315863602 | -0.028306974 |
| CD53 | Triglycerides | 0.922694921 | 0.04 | 0.002321487 | 8.55826E-05 | 0.004557392 |
| FABP1 | Men | 0.684440732 | 0.001 | 0.547002503 | 0.22123232 | 0.872772686 |
| FABP1 | Antilipid R_x_ | 0.678754449 | 0.002 | 0.559038405 | 0.213891059 | 0.90418575 |
| FABP5 | Total Cholesterol:HDL Ratio | 1.148300409 | 0.004 | -0.199500044 | -0.33689775 | -0.062102338 |
| FABP5 | Men | 1.244425893 | 0.01 | -0.315480348 | -0.547335843 | -0.083624854 |
| FABP5 | Age | 1.128999949 | 0.02 | -0.017504538 | -0.032729489 | -0.002279588 |
| FABP5 | Diabetes | 1.27626276 | 0.04 | -0.351925325 | -0.681857807 | -0.021992843 |
| FABP5 | Diastolic Bp | 0.906064332 | 0.04 | 0.01423146 | 0.00058165 | 0.027881271 |
| FGG | Men | 0.65600121 | 0.0005 | 0.608229575 | 0.27676518 | 0.939693969 |
| FGG | Antilipid R_x_ | 0.678515255 | 0.002 | 0.55954687 | 0.208366615 | 0.910727125 |
| FGG | BMI | 0.869723022 | 0.01 | 0.040274412 | 0.009106576 | 0.071442249 |
| FGG | Aspirin 3X/Week | 1.323813319 | 0.02 | -0.404699659 | -0.735332849 | -0.07406647 |
| MRPS26 | Men | 1.606650472 | 0.0005 | -0.684056105 | -0.920395086 | -0.447717124 |
| NR2F6 | Men | 1.692065239 | 0.0005 | -0.758785237 | -1.001255174 | -0.5163153 |
| NR2F6 | BMI | 1.111660004 | 0.01 | -0.030543133 | -0.053342756 | -0.007743509 |
| NR2F6 | Total Cholesterol:HDL Ratio | 1.151421309 | 0.01 | -0.203415771 | -0.347103602 | -0.05972794 |
| NR2F6 | Diastolic Bp | 0.894916534 | 0.03 | 0.0160175 | 0.001742796 | 0.030292205 |
| NR2F6 | Triglycerides | 0.921172142 | 0.04 | 0.002369147 | 0.000134649 | 0.004603645 |
| RPL21 | Men | 1.709206462 | 0.0005 | -0.773326679 | -0.999518211 | -0.547135147 |
| RPL21 | BMI | 1.106335282 | 0.01 | -0.029157725 | -0.050426678 | -0.007888772 |
| RPL21 | Diastolic Bp | 0.89908123 | 0.02 | 0.015347659 | 0.002031297 | 0.02866402 |
| RPL21 | Diabetes | 1.257961392 | 0.04 | -0.331087626 | -0.652960237 | -0.009215014 |
| SERPINA1 | Men | 1.360169888 | 0.001 | -0.443786917 | -0.712000893 | -0.17557294 |
| SERPINA1 | BMI | 1.131448507 | 0.01 | -0.035634184 | -0.060854539 | -0.01041383 |
| SERPINA1 | Age | 1.159897089 | 0.02 | -0.021399687 | -0.039012142 | -0.003787231 |
| SERPINA1 | Diastolic Bp | 0.886387348 | 0.03 | 0.017399078 | 0.001608769 | 0.033189387 |
| VTN | Aspirin 3X/Week | 1.382321119 | 0.002 | -0.467092846 | -0.763585839 | -0.170599854 |
| VTN | Triglycerides | 0.871874392 | 0.005 | 0.003956157 | 0.001216937 | 0.006695376 |

^1^Fold Change for FHS – regression coefficient transformed (2^-β^) to express fold change in gene expression associated with 1 unit change in clinical covariate, with the exception in triglycerides (50 points), systolic and diastolic blood pressure (10 points), age (10 years), and BMI (5 points).

Abbreviations – BMI – Body Mass Index, BP – Blood Pressure, CI – Confidence Interval, HDL – High Density Lipoprotein, R_x_ - Medication
